# The use of strain, strain rate, and displacement by 2D speckle tracking for assessment of systolic left ventricular function in goats: applicability and influence of general anesthesia

**DOI:** 10.1186/s12947-015-0005-8

**Published:** 2015-03-17

**Authors:** Ann-Sabin J Berli, Rahel Jud Schefer, Kathrin Steininger, Colin C Schwarzwald

**Affiliations:** Equine Department, Vetsuisse Faculty, University of Zurich, Winterthurerstrasse 260, Zurich, 8057 Switzerland; Small Animal Department, and Vetsuisse Faculty, University of Zurich, Winterthurerstrasse 260, Zurich, 8057 Switzerland; Farm Animal Department, Vetsuisse Faculty, University of Zurich, Winterthurerstrasse 260, Zurich, 8057 Switzerland

**Keywords:** 2D speckle tracking, Strain, Strain rate, Displacement, Left ventricular function, Goat

## Abstract

**Background:**

Assessment of left ventricular (LV) systolic function can be achieved by conventional echocardiographic methods, but quantification of contractility, regional myocardial function, and ventricular synchrony is challenging. The goal of this study was to investigate the applicability of two-dimensional speckle tracking (2DST) to characterize segmental and global wall motion for assessment of LV function and LV synchrony in healthy goats. We aimed to describe the techniques, report normal values of a variety of 2DST indices, and determine the influence of general anesthesia.

**Methods:**

Prospective study on 22 healthy female Saanen goats (3.7 ± 1.1 y, 60.2 ± 10.5 kg [mean ± SD]). All goats underwent two transthoracic echocardiographic examinations, the first standing and unsedated and the second 7.4 ± 3.5 days later during isoflurane anesthesia and positioned in sternal recumbency. Data analyses were performed offline, blinded, and in random order. Left ventricular longitudinal, radial and circumferential strain and strain rate as well as longitudinal and radial displacement were measured using 2DST methods. Summary statistics were generated and differences of 2DST variables between myocardial segments and treatments (i.e., awake vs. anesthetized) were assessed statistically (alpha level=0.05).

**Results:**

Echocardiographic analyses by 2DST were feasible in all goats and at both time points. Longitudinal systolic strain, strain rate and displacement followed a gradient from apex to base. Absolute systolic strain was generally lower and strain rate was higher in awake goats compared to anesthetized goats. Circumferential and radial indices did not consistently follow a segmental pattern. Generally, peak strain occurred later in anesthetized goats compared to awake goats. General anesthesia did not significantly influence LV synchrony.

**Conclusions:**

2SDT is a valid method for non-invasive characterization of LV wall motion in awake and anesthetized goats. The results of this study add to the understanding of LV mechanical function, aid in the diagnosis of global and segmental LV systolic dysfunction, and will be useful for future cardiovascular studies in this species. However, effects of anesthesia and species-specific characteristics should be considered when goats are used as animal models for human disease.

**Electronic supplementary material:**

The online version of this article (doi:10.1186/s12947-015-0005-8) contains supplementary material, which is available to authorized users.

## Background

The echocardiographic assessment of myocardial function is a pivotal part of every clinical or experimental cardiologic examination, but still presents a considerable challenge for every cardiologist. Traditionally, myocardial function has been assessed by use of two-dimensional (2D), M-Mode, and Doppler echocardiography [1,2]. Although these methods are well established, they serve to assess overall systolic function, but do not provide specific data for quantification of myocardial contractility, regional myocardial function, or ventricular synchrony. At best, conventional echocardiographic methods can be used for subjective visual assessment of regional wall motion or manual tracking of myocardial movement, which requires considerable experience and is highly operator dependent [3-6].

Echocardiographic strain and strain rate imaging using two-dimensional speckle tracking (2DST) has been advocated for assessment of regional and global ventricular function beyond the conventional echocardiographic approach [7-9]. The high sensitivity of this method for early detection of myocardial dysfunction or dyssynchrony related to coronary artery disease, myocardial ischemia, myocardial infarction, or heart failure render this non-invasive diagnostic method suitable for clinical use in humans [4]. It has been used in a variety of species, including humans, dogs, cats, horses, and pigs [4,10-16]. The goat is commonly used as an animal model for the study of human cardiovascular disease [17-20]. Therefore, novel echocardiographic techniques are also pertinent to this species, despite the fact that natural cardiac disease is of minor relevance in goats [21]. However, to our knowledge there are no comprehensive studies investigating the use of strain and strain rate by 2DST in goats.

Therefore, the goal of this study was to investigate the applicability of 2DST to characterize segmental and global wall motion for assessment of LV function and LV synchrony in healthy Saanen goats. We hypothesized that 2DST can be applied to characterize myocardial function in standing, awake goats as well as in anesthetized goats during general anesthesia. We aimed to describe the techniques, report normal values of a variety of 2DST indices, and determine the influence of general anesthesia. We further intended to explore the agreement of the findings of this study to other studies conducted in people and in other animal species to identify similarities and disparities with regard to the physiology of LV mechanics. The results of this study provide fundamental information on the use of 2DST for assessment of LV mechanics in goats and will be useful for future cardiovascular studies in this species.

## Methods

### Study population

22 female Saanen goats aged 3.7 ± 1.1 [mean ± SD] years and with a body weight of 60.2 ± 10.5 kg were studied prospectively. All goats were considered healthy based upon physical examination, cardiac auscultation, and routine echocardiographic examination [1].

None of the goats received medications during the 2 weeks preceding entry into the study. The goats were acclimatized to the hospital for one week before the study. They were housed indoors, kept on straw and had free access to water and hay. Animal experiments were carried out in accordance with the Swiss law on animal protection. All animal studies were approved by the district veterinary office of the Canton of Zurich.

### Study design

All goats underwent a complete echocardiographic examination while standing in a quiet room, unsedated and restrained by an experienced handler. A second echocardiographic examination within 7.4 ± 3.5 days of the first examination was performed immediately following a computed tomography scan conducted within the scope of another investigation, with the goats in general anesthesia and positioned in sternal recumbency. The mean time from induction of anesthesia to the start of the echocardiographic examination was 68 (52–103) min [mean (range)].

### General anesthesia

The animals were fasted for 24 h and deprived of water for 2 h prior to anaesthesia. A 14 G/1.88 in catheter (BD Angiocath, Becton Dickinson, Allschwil, Switzerland) was placed aseptically into the right jugular vein. All animals were premedicated intravenously with 0.1 mg/kg xylazine (Rompun 2%, Provet, Lyssach, Switzerland), diluted in 20 mL of saline (NaCl 0.9%, Braun Medical, Sempach, Switzerland) and given over 5 min with a syringe pump (Syramed μ6000, Arcomed, Regensdorf, Switzerland). Anaesthesia was then induced with either 3 mg/kg racemic ketamine (Narketan 10%, Vetoquinol, Ittigen, Switzerland) or 1.5 mg/kg S-ketamine (Keta-S 6%, Dr. Graeub AG, Bern, Switzerland) i.v. given by manual injection.

Once the animals were recumbent, they were positioned sternally, intubated, and connected to an anesthesia machine. Generally, the goats were allowed to breathe spontaneously throughout the entire duration of anesthesia. Mechanical ventilation was only applied when arterial blood gas analysis revealed a PaCO_2_ > 50 mmHg. In this case, intermittent positive pressure ventilation was used with a tidal volume of 10-15 mL/kg at a rate of 10-15 breaths per minute. Anesthesia was maintained with isoflurane (IsoFlo®, Abbott, Baar, Switzerland) delivered in oxygen and air via a semi-closed circle absorption system with a flow of 60 mL/kg/min during the first 10 min and a flow of 35 mL/kg/min thereafter. The vaporizer was initially set to 2.5% and after 15 min it was reduced and held between 1.5 and 2% to maintain an end-tidal isoflurane concentration of 1.1%. Lactated Ringer’s solution (Ringer-Laktat, Fresenius Kabi, Stans, Switzerland) was administered i.v. at a dose rate of 10 mL/kg/h. During anesthesia, the goats were kept in sternal recumbency.

Each goat was instrumented with an electrocardiogram, a pulse oximeter and an end-tidal gas analyzer. A portable multiparameter monitor (Datex-Ohmeda AS/3 Compact Monitor; Anandic, Schaffhausen, Switzerland) continuously displayed the physiologic monitoring data during the anesthetic period. The ear artery was cannulated for intraarterial blood pressure monitoring and blood sampling for blood gas analyses. Heart rate (HR), respiratory rate, saturation of arterial oxygen, direct arterial blood pressures, end-tidal CO_2_, end-tidal isoflurane, and rectal temperature were monitored. Blood gas analysis was performed at 10 minutes after anesthesia induction and then every 30 minutes for the duration of anesthesia. Following anesthesia, the goats were placed sternally in a padded box. The endotracheal tube was removed once swallowing reflex returned.

### Echocardiography

Transthoracic echocardiography was performed using a high-end digital echocardiograph (GE Vivid 7 Dimension, BTO6, GE Medical Systems, Glattbrugg, Switzerland) with a phased array transducer (M4S, GE Medical Systems, Glattbrugg, Switzerland) at a frequency of 1.9/4.0 MHz (octave harmonics) [22]. A single lead electrocardiogram (lead I) was recorded simultaneously. The lead position was consistent among animals and between repeat echocardiograms. The aortic valve was imaged in 2D grayscale mode in a right parasternal long-axis view of the left ventricular outflow tract (LVOT) and in M-mode in a right parasternal short-axis view, respectively, for subsequent determination of the time of aortic valve closure. The left ventricle (LV) was imaged in 2D mode using a right parasternal four-chamber view optimized to obtain an image of the entire LV at its largest dimensions, including the LV apex. Subsequently, the LV was imaged in three short-axis views, namely at the level of the apex, at the level of the papillary muscles, and at the level of the chordae tendineae. Imaging depth and sector width, respectively, were adjusted to achieve a frame rate > 50 frames/second in 2D imaging mode. Three representative, non-consecutive cardiac cycles were recorded in each view and stored as cine-loops in digital raw data format. Data analyses were performed offline, blinded, and in random order, using a dedicated software package (EchoPAC Software version 6.1.2, GE Medical Systems, Glattbrugg, Switzerland). Three cardiac cycles were analyzed for each imaging plane. For each measured or calculated variable, the average of the three measurements was reported.

The time to aortic valve closure was measured manually (tAVC_m_). It was defined as the time interval between the peak of the electrocardiographic R wave and the closure point of the aortic valve identified on an M-mode recording of aortic valve motion. In cases, in which the closure point of the aortic valve was not clearly identifiable on the M-mode recording, anatomical M-mode was applied to the recorded two-dimensional echo loop of the LVOT, and cursor placement was adjusted to identify the point of aortic valve closure. The corresponding heart rate (HR_AVCm_) for each cycle was calculated as 60,000/R-R interval.

The 2D speckle tracking (2DST) analyses were performed using the 2D Strain module of the analysis software (EchoPAC Software version 6.1.2, GE Medical Systems, Glattbrugg, Switzerland). The 2DST variables were measured as follows: (1) The appropriate long-axis or short-axis image was selected and the Q-Analysis module was started. (2) A single heart cycle was selected by moving the left and the right cursor, respectively, to the peak of the R waves of the electrocardiogram. Each cycle was identified by the image number, the time when the image was recorded, and the first and the last frame of the cycle. The R-R interval was measured and the frame rate was recorded. (3) Subsequently, the 2D Strain module was started. The long-axis (subsequently called LAX) grayscale loops of the LV were analyzed using the “4CH” option, and the short-axis grayscale loops of the LV were analyzed using the “SAX-AP” (apical level, subsequently called SAX-AP), “SAX-PM” (papillary muscle level, subsequently called SAX-PM), and “SAX-MV” option (chordal level, subsequently called SAX-CH), respectively. Once the option had been selected, a region of interest (ROI) was determined by tracing the endocardial border of the LV at end-systole. For the long-axis images, tracing started at the septal mitral valve (MV) annulus and ended at the lateral MV annulus; for the short-axis images, tracing was started at mid-septum and proceeded in a clockwise direction. The papillary muscles were not included in the tracings. The ROI width was adjusted, so that the entire myocardial thickness was covered throughout the cardiac cycle. Subsequently the speckle tracking analysis was started. The software algorithm automatically divided the myocardium into 6 segments, performed the speckle tracking analysis, and provided confirmation of adequate tracking for each segment (Additional file 1: Figure S5). The segments were preselected by the software based on regional wall motion analysis standards applied to human patients and were not adjusted for use in the goat. Hence, apart from slight individual variations, the short-axis segments termed “Sept”, “AntSept” and “Ant” generally depict the interventricular septum, whereas “Lat”, “Post”, and “Inf” depict the cranial, lateral, and caudal LV free wall.

The quality of the tracking was visually assessed by the operator during motion playback. If necessary, the line tracing of the endocardium was adjusted and the speckle tracking analysis was repeated until adequate tracking was achieved. If adequate tracking was not possible despite repeated adjustments of the ROI, another cardiac cycle was chosen for analysis. If the software failed to adequately track a segment even after repeated manual tracing of multiple cycles, the loop was excluded from analysis.

Six curve profiles were obtained corresponding to the average of each myocardial segment (Additional file 1: Figure S6). For the long-axis images (4CH), the following variables were reported by the software: Longitudinal strain (ε_L_), longitudinal strain rate (SR_L_), longitudinal displacement (D_L_), and transverse displacement (D_T_). For the short-axis images (SAX-AP, SAX-PM, SAX-CH), the following variables were reported by the software: Circumferential strain (ε_C_), circumferential strain rate (SR_C_), radial strain (ε_R_), radial strain rate (SR_R_), radial displacement (D_R_). The time of aortic valve closure was automatically calculated and displayed by the software (AVC_a_, Additional file 1: Figure S6).

The measurements for strain, strain rate, and displacement, respectively, were performed on the “Results” screen of the 2D Strain software module. Automated detection of the peak values was verified on the graphical display and corrected as necessary. The mean of the 6 segmental measurements of each variable was calculated to obtain indices of averaged strain, strain rate, and displacement. Strain measurements for each segment included longitudinal, radial, and circumferential peak strain (peak independent of aortic valve closure; termed ε_L_, ε_R_, and ε_C_) as well as peak systolic strain (peak prior to or at the time of aortic valve closure; termed ε_L-sys_, ε_R-sys_, and ε_C-sys_). Where the highest strain value occurred before or at the time of aortic valve closure, peak strain (ε) was identical with peak systolic strain (ε_sys_). Where 2 peaks for strain were present and ε_sys_ was followed by a 2^nd^, higher peak after aortic valve closure, the 2^nd^ peak was considered ε. Where only 1 peak occurring after aortic valve closure was present, ε_sys_ was defined as strain at the time of aortic valve closure and the 2^nd^ peak was termed ε. Post-systolic motion was diagnosed where the highest strain value occurred after automatically determined aortic valve closure (AVC_a_) [7,9,13,14].

Strain rate measurements for each segment included peak systolic strain rate (SR_L-sys_, SR_R-sys_, and SR_C-sys_), peak early-diastolic strain rate (SR_L-E_, SR_R-E_, and SR_C-E_) and peak late-diastolic strain rate (SR_L-A_, SR_R-A_, and SR_C-A_). Displacement measurements for each segment included peak displacement (D_L_, D_T_, D_R_). The R-R interval was measured for each cycle. The time to automatically determined aortic valve closure (tAVC_a_) was calculated as the time interval between the peak of the electrocardiographic R wave and AVC_a_ displayed on the “Trace” screen. The corresponding heart rate (HR_AVCa_) for each cycle was calculated as 60,000/R-R interval.

The time interval from the electrocardiographic R wave to longitudinal, circumferential, and radial peak strain (t_εL_, t_εC_, t_εR_) of each segment was measured in each cycle. The synchrony time index (STI_ε_), a measure of myocardial dyssynchrony, was calculated as the difference in t_ε_ from the earliest to the latest segment [12,13].

### Data analysis and statistics

All statistical and graphical analyses were performed using standard computer software (Microsoft Office Excel 2003, Microsoft Corporation, Redmond, WA; SigmaStat v3.5, SPSS Inc, Chicago, IL; GraphPad Prism v5.00 for Windows, GraphPad Software, San Diego California USA). The agreement between tAVC_a_ and tAVC_m_ was compared by paired t test and Bland-Altman statistics. The corresponding heart rates (HR_AVCm_ and HR_AVCa_) were compared by paired t test. For segmental 2DST indices, 2-way repeated-measures analysis of variance was used to detect differences between segments and treatment (i.e., awake vs. anesthetized). When the F test indicated significant differences, all pairwise multiple comparisons were performed using the Holm-Sidak post hoc test. The effect of general anesthesia on averaged 2DST indices and on STI_ε_ was assessed by paired t tests, reporting the 95% confidence intervals for the difference of means. Validity of the normality assumption was confirmed by assessment of normal probability plots of the residuals. The level of significance was set at P=0.05.

## Results

### Feasibility and quality of recordings

Echocardiographic analyses by 2DST were feasible in all 22 goats and at both time points (i.e., awake, anesthetized). Accidentally, in one goat the LAX and in another one the SAX-CH images had not been recorded.

The frame rate of the recordings ranged between 70.2 and 103.8 frames per seconds (85.1 ± 2.6 [mean ± SD]). Tracking was considered inaccurate in 1,869 of total 31,512 measured segments (5.93%) based on automated verification and visual assessment of tracking by the operator (LAX, 178 segments; SAX-AP, 488 segments; SAX-PM, 724 segments; SAX-CH, 479 segments).

### AVC and HR

There was no significant difference between tAVC_a_ and tAVC_m_ except for measurements obtained at the apical short-axis level during anesthesia and at the chordal short-axis level in awake and anesthetized goats (Table 1). Generally, tAVC_a_ occurred later at the chordal level compared to the papillary muscle and the apical level, respectively. Mean bias was lowest for long-axis analyses and short-axis analyses at the papillary muscle level.

**Table 1:** **Agreement between manually measured and automatically determined time of aortic valve closure and corresponding heart rates**

**View**	**Treatment**	**tAVC**_**m**_ **(ms) [mean ± SD]**	**tAVC**_**a**_ **(ms) [mean ± SD]**	**Bias (ms)**	**95% LoA (ms)**	**95% CI for difference of means (ms)**	**p value**	**HR**_**AVCm**_ ** (min** ^**−1**^ **) [mean ± SD]**	**HR**_**AVCa**_ **(min** ^**−1**^ **) [mean ± SD]**	**95% CI for difference of means (min** ^**−1**^ **)**	**p value**
	Awake	281.6 ± 23.5						98 ± 19.6			
	Anesthetized	335.8 ± 24.4						85 ± 11.1			
LAX	Awake		285.68 ± 27.57	−4	−33 to +25	−10.53 to 2.24	0.192		95 ± 20.39	−1.1 to +6.6	0.157
LAX	Anesthetized		331.11 ± 22.9	+6	−30 to +43	−2.38 to 14.60	0.149		84 ± 10.46	−0.3 to +1.3	0.186
SAX-AP	Awake		274.59 ± 30.36	+5	−22 to +32	−1.11 to 11.17	0.103		99 ± 21.92	−4.1 to +3.3	0.812
SAX-AP	Anesthetized		323.97 ± 24.18	+12	−31 to +55	2.07 to 21.66	*0.020*		85 ± 10.97	−0.8 to +0.7	0.960
SAX-PM	Awake		284.79 ± 31.13	−4	−50 to +41	−14.33 to 5.75	0.385		97 ± 23.46	−4.7 to +5.8	0.823
SAX-PM	Anesthetized		330.78 ± 18.89	+5	−34 to +44	−3.85 to 13.95	0.251		85 ± 11.02	−0.5 to +0.8	0.625
SAX-CH	Awake		311.14 ± 24.26	−30	−66 to +7	−38.11 to −21.55	*<0.0001*		96 ± 19.71	−1.9 to +6.4	0.277
SAX-CH	Anesthetized		346.41 ± 25.35	−11	−52 to +31	−20.04 to −1.109	*0.030*		85 ± 10.83	−1.0 to +0.8	0.825

LAX, long axis view; SAX-AP, short axis view at apical level; SAX-PM, short axis view at papillary muscle level; SAX-CH, short axis view at chordal level; tAVC_m_, manually measured time of aortic valve closure based on M-mode recordings of aortic valve motion; tAVC_a_, automatically determined time of aortic valve closure based on 2DST-based strain analyses; HR_AVCm_, heart rate derived from cardiac cycles used for measurement of tAVC_m_; HR_AVCa_, heart rate derived from cardiac cycles used for measurement of tAVC_a_; LoA, limits of agreement; CI, confidence interval.

No significant differences were identified between HR_AVCa_ and HR_AVCm_ in any of the views and treatments.

### Post-systolic motion

**Figure 1:**
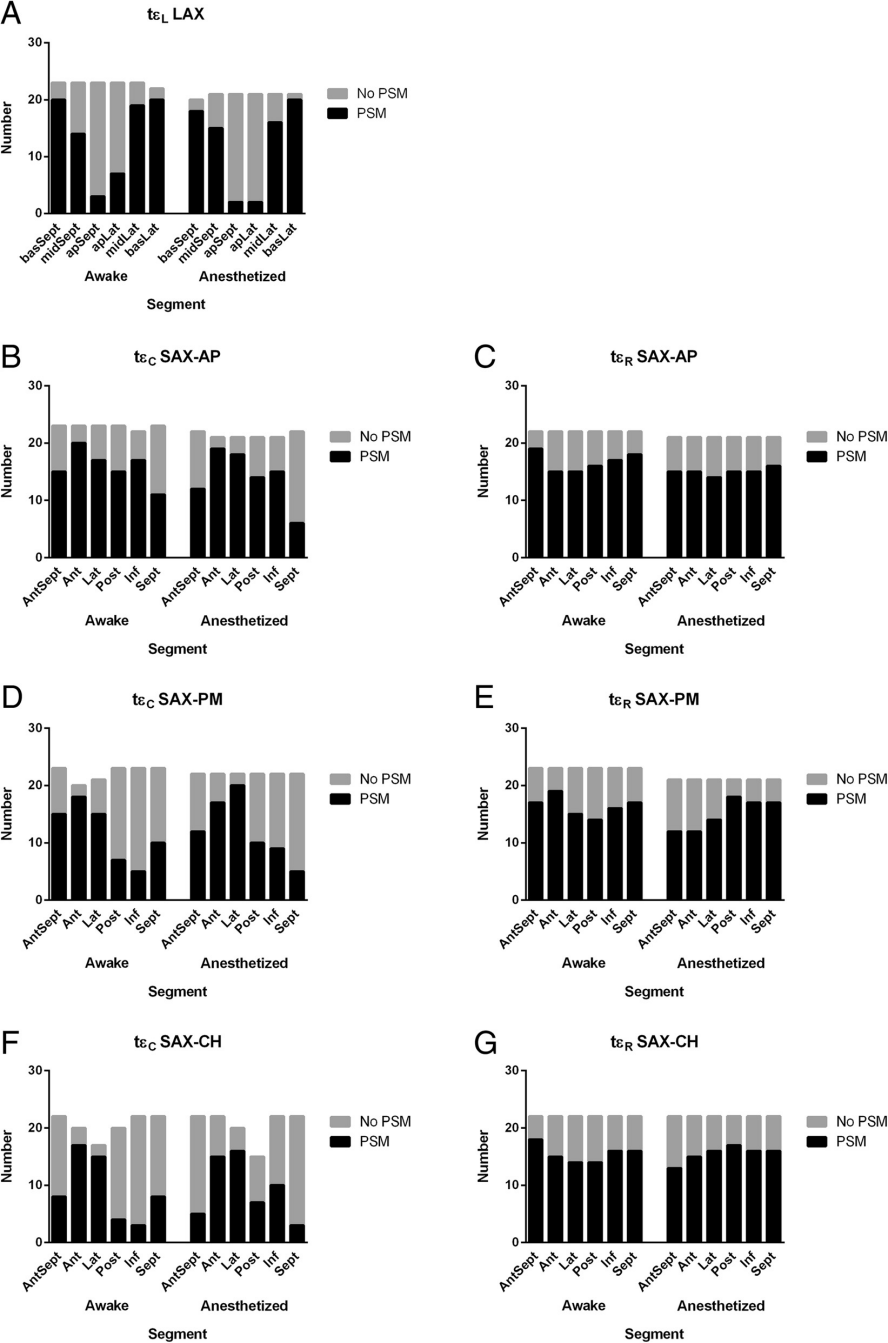
**Post-systolic motion in left ventricular long-axis and short-axis recordings.** Prevalence of post-systolic motion (PSM) in awake and anesthetized goats. Post-systolic motion was diagnosed where peak strain occurred after aortic valve closure. **A**: PSM based on timing of longitudinal peak strain (tε_L_), **B**: PSM based on timing of circumferential peak strain (t_εC_) at the apical short-axis level (SAX-AP), **C**: PSM based on timing of radial peak strain (t_εR_) at the apical short-axis level (SAX-AP), **D**: PSM based on t_εC_ at the papillary muscle short-axis level (SAX-PM), **E**: PSM based on _tεR_ at the papillary muscle short-axis level (SAX-PM), **F**: PSM based on t_εC_ at the chordal short-axis level (SAX-CH), **G**: PSM based on t_εR_ at the chordal short-axis level (SAX-CH).

The occurrence of post-systolic motion is summarized in Figure 1. Peak longitudinal strain occurred before or at AVC in the apical segments of most animals, whereas post-systolic motion, with t_εL_ occurring after AVC, was more common in mid and basal segments. In circumferential direction, the occurrence of post-systolic motion differed depending on imaging plane and myocardial segments. Finally, in radial direction, post-systolic motion was seen in the majority of animals in all three imaging planes.

### Averaged strain and strain rate

Heart rate, arterial blood pressure, averaged strain and strain rate values in awake and anesthetized goats, respectively, are presented in Table 2. The difference in heart rate between awake and anesthetized goats did not reach statistical significance. Absolute strain values of awake goats were generally lower compared to those in anesthetized goats, but not all differences reached statistical significance. Conversely, absolute systolic strain rate values in awake goats were all significantly higher than those recorded under anesthesia, except SR_R-sys_ at the SAX-AP level. No clear trend was detected for early-diastolic strain rate, whereas absolute values for late-diastolic strain rate were generally lower during anesthesia compared to the awake state.

**Table 2:** **Averaged strain and strain rate of the left ventricle obtained in different imaging planes in awake and anesthetized goats**

**Variable**	**Units**	**Awake (mean ± SD)**	**Anesthetized (mean ± SD)**	**95% CI for difference of means**	**p value**
HR	1/min	94 ± 20	85 ± 11	−2 to 18	0.103
MAP	mmHg	n/a	87 ± 18	n/a	n/a
**LAX**					
ε_L_	%	−27.44 ± 2.22	−28.65 ± 2.21	0.28 to 2.15	*0.014*
ε_L-sys_	%	−26.17 ± 2.23	−27.17 ± 2.49	−0.13 to 2.13	0.080
SR_L-sys_	1/s	−2.29 ± 0.36	−1.96 ± 0.30	−0.51 to −0.15	*0.001*
SR_L-E_	1/s	2.92 ± 0.60	3.11 ± 0.35	−0.56 to 0.17	0.276
SR_L-A_	1/s	1.92 ± 0.81	1.50 ± 0.47	0.02 to 0.82	*0.039*
**SAX-AP**					
ε_C_	%	−23.11 ± 2.17	−25.58 ± 3.11	0.96 to 3.98	*0.003*
ε_C-sys_	%	−22.80 ± 2.45	−25.10 ± 3.21	0.75 to 3.84	*0.006*
ε_R_	%	58.58 ± 7.97	58.41 ± 11.65	−7.03 to 6.48	0.932
ε_R-sys_	%	55.24 ± 8.94	55.59 ± 12.65	−8.06 to 6.53	0.828
SR_C-sys_	1/s	−2.15 ± 0.37	−1.9 ± 0.42	−0.48 to −0.03	*0.028*
SR_C-E_	1/s	2.93 ± 0.59	3.24 ± 0.62	−0.66 to 0.53	0.092
SR_C-A_	1/s	1.49 ± 0.89	1.08 ± 0.49	0.01 to 0.80	*0.047*
SR_R-sys_	1/s	2.36 ± 0.34	2.14 ± 0.49	−0.04 to 0.47	0.088
SR_R-E_	1/s	−2.74 ± 0.77	−2.81 ± 0.74	−0.42 to 0.47	0.921
SR_R-A_	1/s	−2.18 ± 1.39	−1.38 ± 0.80	−1.30 to −0.16	*0.015*
**SAX-PM**					
ε_C_	%	−22.98 ± 2.87	−24.64 ± 2.92	0.09 to 3.24	*0.040*
ε_C-sys_	%	−22.35 ± 2.97	−23.96 ± 3.23	−0.07 to 3.29	0.060
ε_R_	%	58.44 ± 9.21	63.92 ± 15.25	−14.71 to 2.22	0.139
ε_R-sys_	%	55.26 ± 9.95	61.86 ± 15.67	−14.74 to 1.45	0.102
SR_C-sys_	1/s	−2.07 ± 0.32	−1.86 ± 0.3	−0.39 to −0.04	*0.016*
SR_C-E_	1/s	3.16 ± 0.49	3.20 ± 0.39	−0.33 to 0.25	0.783
SR_C-A_	1/s	1.28 ± 0.51	1.09 ± 0.45	−0.08 to 0.47	0.162
SR_R-sys_	1/s	2.50 ± 0.48	2.21 ± 0.29	0.06 to 0.50	*0.016*
SR_R-E_	1/s	−2.72 ± 0.53	−2.67 ± 0.67	−0.40 to 0.34	0.869
SR_R-A_	1/s	−1.83 ± 0.72	−1.38 ± 0.54	−0.81 to −0.03	*0.038*
**SAX-CH**					
ε_C_	%	−22.12 ± 3.05	−25.43 ± 3.03	−5.26 to −1.41	*0.002*
ε_C-sys_	%	−14.19 ± 1.94	−24.84 ± 3.24	−12.53 to −8.88	*<0.001*
ε_R_	%	57.71 ± 10.31	63.36 ± 12.53	−0.69 to 14.12	0.073
ε_R-sys_	%	53.90 ± 9.62	59.80 ± 12.88	0.33 to 14.04	*0.041*
SR_C-sys_	1/s	−2.11 ± 0.48	−1.82 ± 0.21	0.11 to 0.48	*0.004*
SR_C-E_	1/s	3.09 ± 0.62	3.34 ± 0.50	−0.06 to 0.60	0.101
SR_C-A_	1/s	1.32 ± 0.43	1.19 ± 0.46	−0.40 to 0.18	0.420
SR_R-sys_	1/s	2.60 ± 0.43	2.33 ± 0.26	−0.55 to −0.03	*0.031*
SR_R-E_	1/s	−3.05 ± 0.74	−2.79 ± 0.73	−0.19 to 0.61	0.290
SR_R-A_	1/s	−1.71 ± 0.60	−1.59 ± 0.57	−0.27 to 0.44	0.617

HR, heart rate; MAP, mean arterial pressure; LAX, long axis view; SAX-AP, short axis view at apical level; SAX-PM, short axis view at papillary muscle level; SAX-CH, short axis view at chordal level; ε_L_, longitudinal peak strain; ε_L-sys_, longitudinal peak systolic strain; SR_L-sys_, longitudinal peak systolic strain rate; SR_L-E_, longitudinal peak early-diastolic strain rate; SR_L-A_, longitudinal peak late-diastolic strain rate; ε_C_, circumferential peak strain; ε_C-sys_, circumferential peak systolic strain; ε_R_, radial peak strain; ε_R-sys_, radial peak systolic strain; SR_C-sys_, circumferential peak systolic strain rate; SR_C-E_, circumferential peak early-diastolic strain rate; SR_C-A_, circumferential peak late-diastolic strain rate; SR_R-sys_, radial peak systolic strain rate; SR_R-E_, radial peak early-diastolic strain rate; SR_R-A_, radial peak late-diastolic strain rate.

Absolute values for peak systolic strain (ε_sys_) were significantly lower than those for peak strain (ε), except for circumferential strain in awake goats recorded at the SAX-AP level (Table 3).

**Table 3:** **Comparison between averaged peak strain and averaged peak systolic strain**

**Direction of strain**	**Imaging plane**	**Treatment**	**Peak strain, ε (%) [mean ± SD]**	**Peak systolic strain, ε**_**sys**_ **(%) [mean ± SD]**	**95% CI for difference of means**	**p value**
Longitudinal	LAX	Awake	−27.44 ± 2.22	−26.17 ± 2.23	−1.56 to −0.98	*<0.001*
		Anesthetized	−28.65 ± 2.21	−27.17 ± 2.49	−1.91 to −1.06	*<0.001*
Circumferential	SAX-AP	Awake	−23.22 ± 2.17	−22.80 ± 2.45	−0.67 to 0.05	0.087
		Anesthetized	−25.58 ± 3.11	−25.10 ± 3.21	−0.71 to −0.26	*<0.001*
	SAX-PM	Awake	−22.98 ± 2.87	−22.35 ± 2.97	−0.89 to −0.37	*<0.001*
		Anesthetized	−24.64 ± 2.92	−23.96 ± 3.23	−1.01 to −0.35	*<0.001*
	SAX-CH	Awake	−22.12 ± 3.05	−14.19 ± 1.94	−8.78 to −7.09	*<0.001*
		Anesthetized	−25.43 ± 3.03	−24.84 ± 3.24	−0.76 to −0.40	*<0.001*
Radial	SAX-AP	Awake	58.58 ± 7.97	55.24 ± 8.94	2.31 to 4.36	*<0.001*
		Anesthetized	58.41 ± 11.65	55.59 ± 12.65	1.96 to 3.67	*<0.001*
	SAX-PM	Awake	58.44 ± 9.21	55.26 ± 9.59	2.31 to 4.02	*<0.001*
		Anesthetized	63.92 ± 15.25	61.86 ± 15.67	1.25 to 2.88	*<0.001*
	SAX-CH	Awake	57.71 ± 10.31	53.90 ± 9.62	2.75 to 4.86	*<0.001*
		Anesthetized	63.36 ± 12.53	59.80 ± 12.88	2.50 to 4.62	*<0.001*

LAX, long axis view; SAX-AP, short axis view at apical level; SAX-PM, short axis view at papillary muscle level; SAX-CH, short axis view at chordal level.

### Segmental strain, strain rate, and displacement

**Figure 2:**
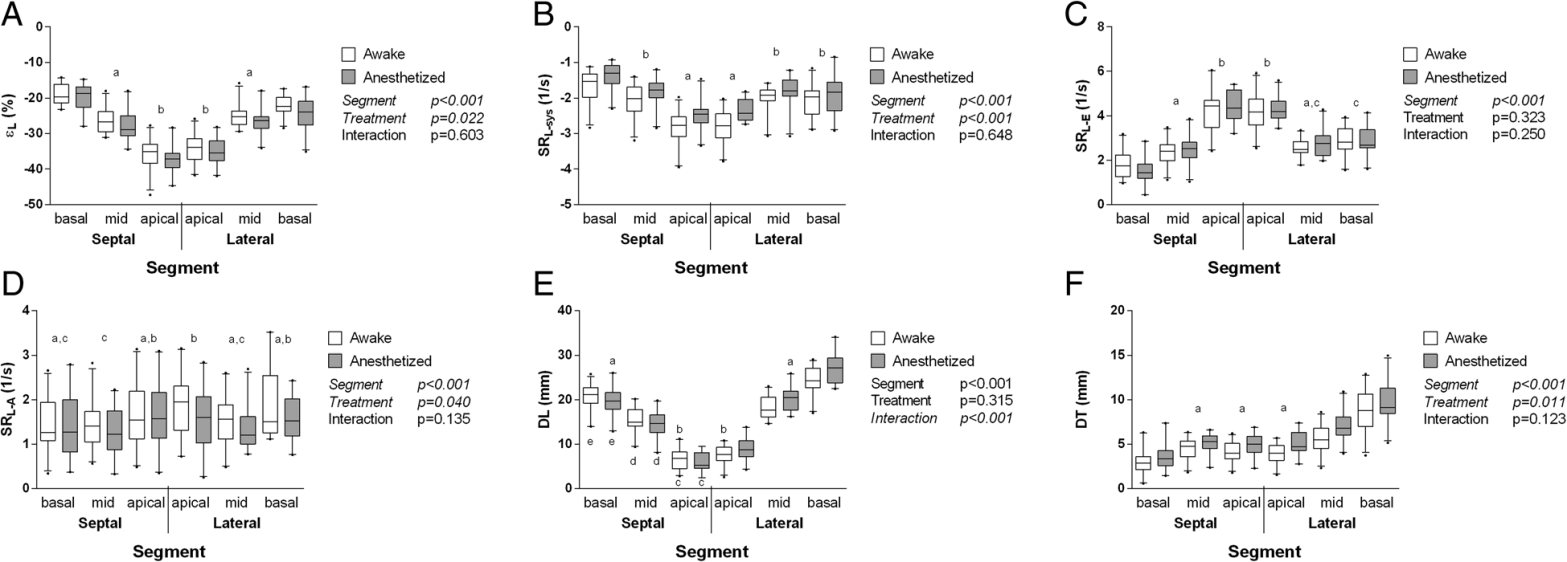
**Segmental 2DST analyses of left ventricular long-axis recordings.** **A**: ε_L_, longitudinal peak strain. **B**: SR_L-sys_, longitudinal peak systolic strain rate. **C**: SR_L-E_, longitudinal peak early-diastolic strain rate. **D**: SR_L-A_, longitudinal peak late-diastolic strain rate. **E**: D_L_, longitudinal peak displacement. **F**: D_T_, transverse peak displacement. Box-and-whisker diagrams, with the line near the middle of the box indicating the median, the top and the bottom of the box indicating the upper and lower quartile, and the whiskers indicating the 5^th^ and 95^th^ percentile observations, respectively. P values of the F test are listed next to each graph; factors for which multiple comparison post hoc testing was performed are displayed in italics. Segments and treatments marked with the same letter were not significantly different from each other when undergoing post hoc testing for multiple comparisons.

**Figure 3:**
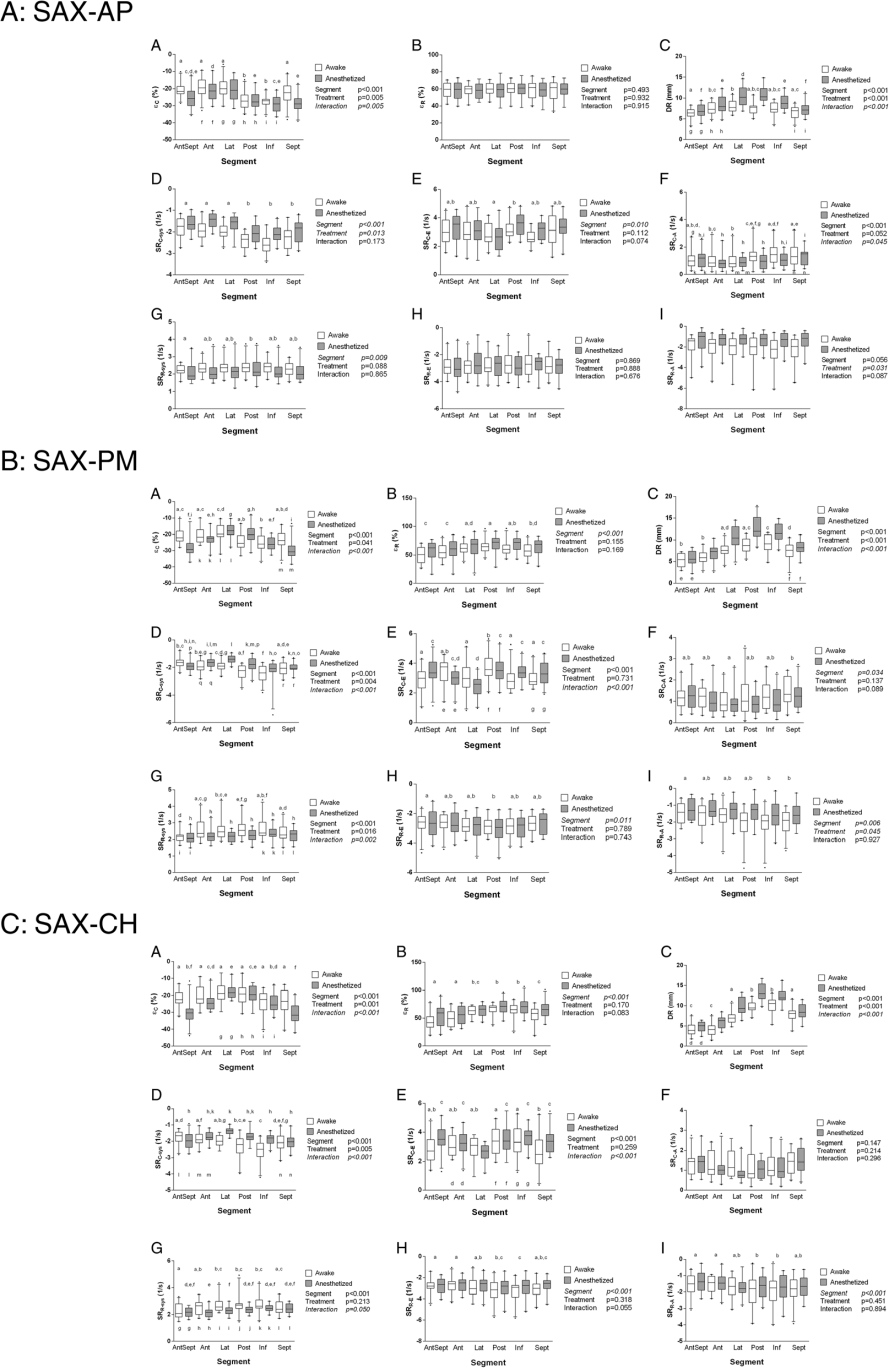
**A-C. Segmental 2DST analyses of left ventricular short-axis recordings at the apical level (A), at the papillary muscle level (B) and at the chordal level (C).** **A**: ε_C_, circumferential peak strain. **B**: ε_R_, radial peak strain. **C**: DR, radial peak displacement. **D**: SR_C-sys_, circumferential peak systolic strain rate. **E**: SR_C-E_, circumferential peak early-diastolic strain rate. **F**: SR_C-A_, circumferential peak late-diastolic strain rate. **G**: SR_R-sys_, radial peak systolic strain rate. **H**: SR_R-E_, radial peak early-diastolic strain rate. **I**: SR_R-A_, radial peak late-diastolic strain rate. Box-and-whisker diagrams, with the line near the middle of the box indicating the median, the top and the bottom of the box indicating the upper and lower quartile, and the whiskers indicating the 5^th^ and 95^th^ percentile observations, respectively. P values of the F test are listed next to each graph; factors for which multiple comparison post hoc testing was performed are displayed in italics. Segments and treatments marked with the same letter were not significantly different from each other when undergoing post hoc testing for multiple comparisons.

Segmental analyses are presented in Figures 2 and 3 and in Additional file 1: Table S5. In LAX (Figure 2), there was a gradient in ε_L_ from apex to the base, with the largest strain at the apex and the lowest at the base. Furthermore, anesthesia significantly influenced ε_L_ independent of segment, with absolute values for ε_L_ being higher during anesthesia than in awake goats. Similarly, SR_L-sys_ significantly differed between segments (highest strain rate in apical segments) and between treatments (higher absolute strain rate in awake compared to anesthetized goats, independent of segment). SR_L-E_ significantly differed between segments (highest strain rate in apical segments) but not between treatments. SR_L-A_ significantly differed between segments (without a clear gradient) and between treatments (higher strain rate in awake compared to anesthetized goats, independent of segment). Longitudinal displacement differed significantly between segments (with the largest displacement in basal segments and the smallest in apical segments) and between treatments (with varying effect of anesthesia depending on segment). Transverse displacement differed significantly between segments (with an apparent gradient from lateral basal over apical to septal basal segments) and between treatments (higher displacement in anesthetized compared to awake goats).

In SAX views (Figure 3), ε_C_ significantly differed between segments and treatments (with varying effect of anesthesia depending on segment) and with an apparent gradient in anesthetized goats at PM and CH levels. At PM and CH levels, ε_R_ significantly differed between segments (without a clear gradient) but not between treatments. DR significantly differed between segments (with highest values in LVFW segments) and between treatments. SR_C-sys_ differed significantly between segments and treatments (with effect of anesthesia depending on segment, except at AP level). SR_C-E_ differed between segments at all levels (without an obvious gradient), with a segmental effect of anesthesia at PM and CH levels. At AP level, SR_C-A_ differed significantly between segments (without an obvious gradient), with varying segmental effects of anesthesia. SR_R-sys_ significantly differed between segments (without an obvious gradient), with a segmental effect of anesthesia at the PM and CH level. SR_R-E_ differed significantly between segments at the PM and CH, but not the AP level. Finally, absolute SR_R-A_ was significantly higher in the awake state at the AP and PM level, and it differed between segments at the PM and the CH level.

### Segmental timing of peak strain

**Figure 4:**
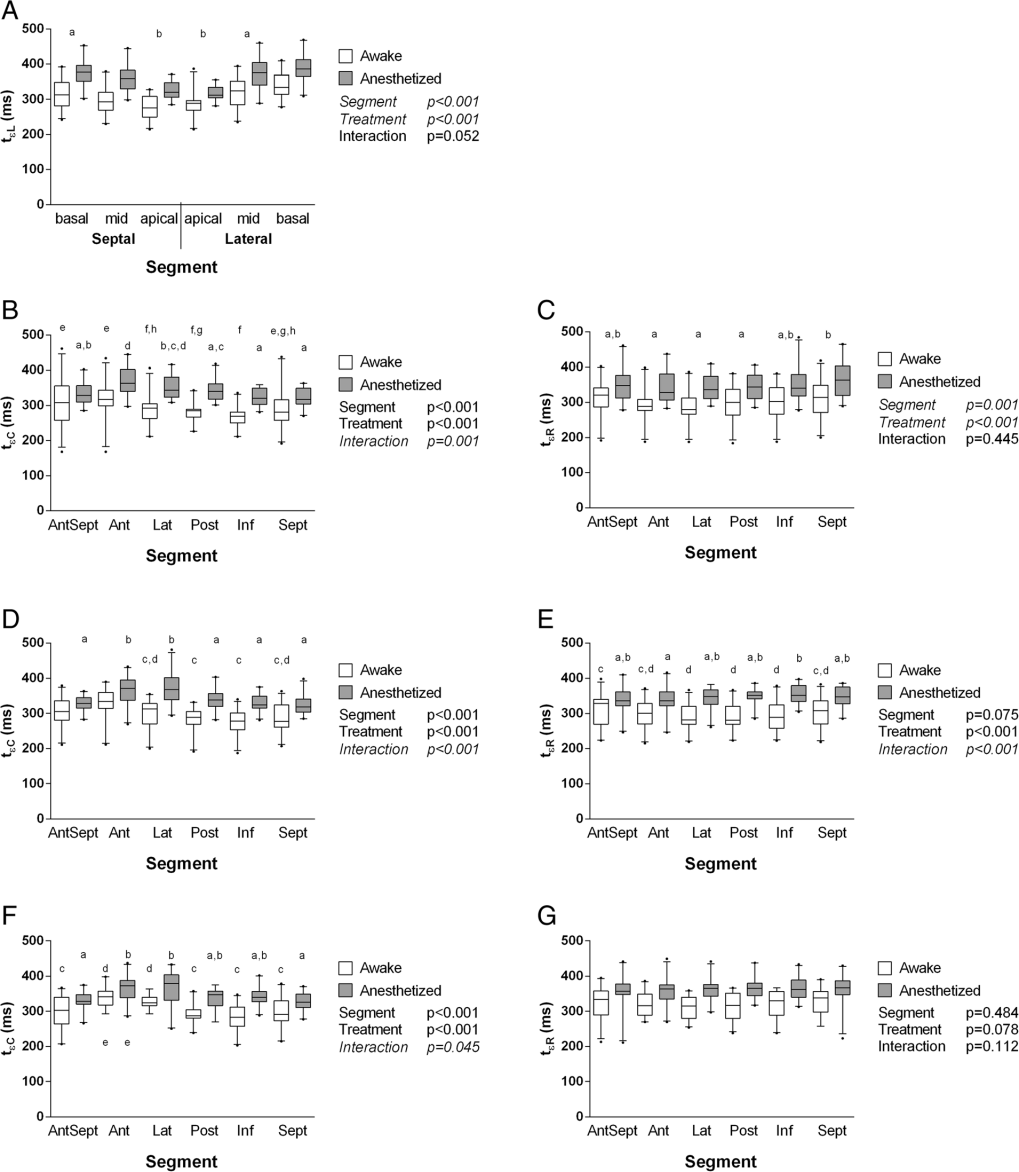
**Segmental timing of peak strain in left ventricular long-axis and short-axis recordings.** Segmental timing of peak strain, expressed as the time interval from the electrocardiographic R wave to longitudinal, circumferential, and radial peak strain of each segment. **A**: Time to longitudinal peak strain (t_εL_) in left ventricular long-axis recordings. **B**: Time to circumferential peak strain (t_εC_) in left ventricular short-axis recordings at the apical level. **C**: Time to radial peak strain (t_εR_) in left ventricular short-axis recordings at the apical level. **D**: Time to circumferential peak strain (t_εC_) in left ventricular short-axis recordings at the papillary muscle level. **E**: Time to radial peak strain (t_εR_) in left ventricular short-axis recordings at the papillary muscle level. **F**: Time to circumferential peak strain (t_εC_) in left ventricular short-axis recordings at the chordal level. **G**: Time to radial peak strain (t_εR_) in left ventricular short-axis recordings at the chordal level. Box-and-whisker diagrams, with the line near the middle of the box indicating the median, the top and the bottom of the box indicating the upper and lower quartile, and the whiskers indicating the 5^th^ and 95^th^ percentile observations, respectively. P values of the F test are listed next to each graph; factors for which multiple comparison post hoc testing was performed are displayed in italics. Segments and treatments marked with the same letter were not significantly different from each other when undergoing post hoc testing for multiple comparisons.

Segmental timing of peak strain values (t_εL_, t_εC_, t_εR_) are presented in Figure 4A-G and in Additional file 1: Table S6. Generally, peak strain occurred significantly later in anesthetized goats compared to awake goats. Longitudinal peak strain occurred slightly earlier in apical segments compared to mid and basal segments. Circumferential peak strain generally occurred later in segments “Ant” and “Lat” in the anesthetized goats (although not all SAX-levels reached the level of significance), whereas segmental differences in radial peak strain did not follow an obvious pattern.

### Synchrony time index

There were no significant differences between synchrony time indices between anesthetized and awake goats (Table 4).

**Table 4:** **Synchrony time index**

**Imaging plane variable**	**Units**	**Awake [mean ± SD]**	**Anesthetized [mean ± SD]**	**95% CI for difference of means**	**p value**
**LAX**					
STI_εL_	ms	90.28 ± 14.23	97.98 ± 19.06	−19.46 to 2.97	0.141
**SAX-AP**					
STI_εC_	ms	93.08 ± 31.41	81.18 ± 33.65	−6.37 to 30.16	0.190
STI_εR_	ms	58.83 ± 36.53	73.42 ± 52.91	−47.87 to 17.59	0.345
**SAX-PM**					
STI_εC_	ms	81.89 ± 25.97	86.20 ± 30.04	−18.36 to 9.75	0.531
STI_εR_	ms	50.55 ± 22.37	50.14 ± 27.41	−16.33 to 16.00	0.983
**SAX-CH**					
STI_εC_	ms	93.06 ± 32.40	91.17 ± 32.78	−19.40 to 23.52	0.847
STI_εR_	ms	64.32 ± 47.00	57.27 ± 42.52	−19.08 to 42.98	0.431

LAX, long axis view; SAX-AP, short axis view at apical level; SAX-PM, short axis view at papillary muscle level; SAX-CH, short axis view at chordal level; STI_εL_, Synchrony time index based on longitudinal peak strain; STI_εC_, Synchrony time index based on circumferential peak strain; STI_εR_, Synchrony time index based on radial peak strain.

## Discussion

The present study shows that 2DST is applicable to characterize LV wall motion in goats in an awake, standing position and in sternal recumbency under general anesthesia.

### Aortic valve closure and post-systolic motion

The time interval between the electrocardiographic R wave and AVC reflects the duration of the electromechanical systole. Since positioning of the ECG leads might influence QRS conformation and thereby the ability to depict the true onset of electrical events in the ventricle, consistent ECG lead placement is important. Automatically determined tAVC_a_ may be less susceptible to variability compared to manually measured tAVC_m_ using M-mode recordings, because the aortic valve is moving relative to the M-mode cursor line during the cardiac cycle [8]. Furthermore, automated timing of aortic valve closure on each cardiac cycle may in fact be superior to an averaged estimation of tAVC_m_, as the latter cannot account for variations of heart rate between different cycles [23]. However, relative bias between tAVC_a_ and tAVC_m_ can differ depending on imaging plane. The findings of this study are consistent with previous findings in horses [16], indicating that tAVC_a_ occurs later at the chordal level compared to the papillary muscle and the apical level, respectively, and that mean bias is small in the long-axis plane (Table 1). However, even the largest relative mean bias (for awake recordings at the chordal level in a short-axis view) was less than 10% and might not be clinically relevant. Therefore, we suggest that automated tAVC_a_ can be readily used for routine 2DST analyses in goats.

The timing of AVC may become important for identification of post-systolic myocardial motion, which is reported to occur in > 30% of myocardial segments in normal human subjects, but may be pathologic if there is a concomitant reduction in systolic strain, especially if the post-systolic thickening is marked [7,9,24]. In previous studies in healthy horses, post-systolic myocardial motion was present in 15 of 16 horses, affecting 31–96% of myocardial segments [13,15,16]. Similarly, the high prevalence of post-systolic motion in this study suggests that this phenomenon might be physiological in goats (Figure 1), with absolute peak strain values (independent of AVC) generally being higher than peak systolic strain values occurring before or at AVC (Table 3). However, no attempts were made to grade the degree of post-systolic motion or to investigate its causes or its clinical relevance. Therefore, it remains uncertain whether the definition of post-systolic motion used in this study would be clinically useful and relevant.

### Averaged strain and strain rate

Myocardial function during the heart cycle is determined by preload, contractility, afterload, and heart rate, which are all influenced by autonomic traffic [25,26]. Traditional echocardiographic ejection phase indices of systolic LV function generally do not reflect contractility per se, since they are also influenced by loading, rate, and rhythm. Peak strain correlates well with stroke volume and ejection fraction and is therefore also influenced by changes in loading conditions, contractility, and heart rate [6,8]. In this study, heart rate during anesthesia was slightly lower compared to heart rate in awake goats, although the difference was not statistically significant. However, the relationship between strain and conventional indices of LV function was not assessed and the study design did not allow detailed investigation of the influence of loading conditions and heart rate. Longitudinal and circumferential strain measurements were generally higher in anesthetized compared to awake goats, consistent with an overall increase in systolic LV performance. These findings were in agreement with previously reported findings obtained from the same population of goats, indicating that M-mode-based and 2DE-based LV ejection phase indices and peak systolic LV wall thickness were significantly higher under general anesthesia [22].

Peak systolic strain rate seems to be more resistant against changes in heart rate and loading conditions and more closely reflects myocardial contractility [5,7,8]. In this study, the lower peak systolic strain rate values in anesthetized compared to awake goats therefore suggest depressed myocardial contractility, which could be explained by negative inotropic drug effects and decreased sympathoadrenergic activity in anesthetized goats. However, overall LV systolic performance appeared to be increased (see above). This can be attributed to the sum of interacting effects of altered loading, contractility, and heart rate caused by anesthetic drugs, fluid therapy, and mechanical ventilation [22].

Overall, longitudinal and circumferential peak strain and strain rate appear most sensitive to detect differences between awake and anesthetized goats, suggesting that these variables best be used for assessment of LV systolic function in goats.

Strain rate measurements can also be applied for assessment of LV diastolic function. Early-diastolic strain rate (SR_E_) is determined by active LV relaxation, LV compliance, and filling pressures [8]. Late-diastolic strain rate (SR_A_) is related to active atrial contraction (determined by atrial contractility, preload, and afterload) and LV compliance at end-diastole [8]. In this study, SR_E_ was not significantly different in anesthetized compared to awake goats, suggesting that LV relaxation and filling was not markedly altered by anesthesia or that 2DST is not sensitive enough to detect slight alterations. Conversely, SR_A_ was higher in awake goats in longitudinal as well as radial (apical and papillary muscle level) and circumferential (apical level) direction, consistent with a slight depression of active atrial contraction in the anesthetized state. This was in agreement with previous findings obtained on the same population of goats, indicating that active left-atrial contraction was slightly lower in anesthetized goats [22].

### Segmental strain, strain rate and displacement

Segmental 2DST indices represent regional contractile function and are used for detection of abnormal regional wall motion patterns and ventricular dyssynchrony and in cardiac resynchronization therapy in humans [27].

In this study, longitudinal strain, systolic strain rate, and early-diastolic strain rate decreased gradually from apex to base (Figure 2). This suggests that the apical myocardial segments of the LV deform more dynamically compared to the mid-wall and basal segments, which is in agreement with findings in previous experimental studies and in human and horse studies [14,16,28-30]. The gradual increase in longitudinal displacement from apex to base (Figure 2E) is consistent with the fact that the mitral annulus is pulled down toward the apex during the ejection phase [16,28].

In SAX imaging planes, segmental differences and effects of anesthesia were generally more pronounced in the circumferential compared to the radial direction. The higher radial strain, strain rate, and displacement in the “Lat”, “Post”, “Inf”, and “Sept” segments in the SAX-PM and SAX-CH planes indicate more pronounced systolic thickening and motion of the LV free wall compared to the septum. These findings also correspond to a previous study in horses [15].

Overall, effects of anesthesia corresponded to those described above for averaged strain and strain rate values, suggesting improved overall systolic LV function in face of depressed myocardial contractility. Effects of anesthesia on radial displacement were in agreement with previously reported 2DE and M-mode indices of LV systolic function in the same population of goats [22].

The timing of segmental peaks strain describes the propagation of mechanical activation. In agreement with previous studies [28,31], maximum longitudinal LV deformation in this study occurred significantly earlier at the apex compared to mid ventricular and basal segments (Figure 4A), consistent with the fact that myocardial depolarization begins near the apical septum and spreads toward the base [32].

In SAX imaging planes, timing of circumferential peak strain values showed more segmental variation compared to radial strain values (Figure 4B-G), possibly related to the helical arrangement of the myofibers. Cranial and lateral segments (“Ant”, “Lat”) generally showed a delayed peak activation compared to other segments. This has also been observed in horses [15].

During anesthesia, peak strain generally was delayed, suggesting that ejection time was prolonged. This can be attributed to the anesthesia-induced decrease in heart rate (Table 1), combined with altered contractility and loading conditions [1]. No attempts were made to correct segmental peak timing for differences in heart rate.

There is no single synchrony index that is preferred for assessment of ventricular dyssynchrony in people. Studies suggest that combining dyssynchrony data from different methods may be of additive value [33]. In this study, we chose to investigate a synchrony time index that can easily be calculated based on the maximum differences in timing of peak strain [12,27]. Based on the STIs, ventricular synchrony appeared higher in radial compared to longitudinal and circumferential direction and was not influenced by general anesthesia. However, other studies in horses [13,14] and humans [9,34] indicated that reliability of the STI was insufficient for clinical use. Therefore, the effect of anesthesia on ventricular synchrony in goats may not be conclusively assessed based on the present data and further studies are needed to investigate the clinical relevance and the best diagnostic approach to LV dyssynchrony in goats.

Acquisition of 2DE images for assessment of longitudinal motion of the LV in large animals such as adult goats is limited by the fact that apical long-axis views cannot be obtained because of anatomical constraints. While the 2DST software algorithm used in this study is able to correctly track longitudinal myocardial motion independent of image orientation (personal communication, manufacturer’s application specialist), spatial resolution is slightly reduced when the longitudinal axis of the heart is perpendicular to the ultrasound beam. Therefore it is important to standardize imaging planes when comparing studies between individuals or over time and when comparing individual measurements to normal reference intervals.

Since only female goats were included in this study, the influence of sex on 2DST variables could not be assessed. Regression analyses were not able to detect any significant relationship of 2DST variables to age and body weight, respectively (data not shown), but the study population was relatively homogenous and the range of available ages and body weights was narrow.

A true limitation of this study is the lack consideration of alterations in blood pressures between the awake and the anesthetized state, since loading conditions might significantly influence strain variables. However, the current study setting did not allow invasive measurements of arterial blood pressures in awake goats.

Also, this study was not designed to comprehensively assess the repeatability and reproducibility of recordings and measurements. Therefore, while significant differences between segments and treatments, respectively, can be established on a population level, the diagnostic validity for detection of subtle changes in strain and strain rate in individual goats in a clinical setting is unknown to date.

## Conclusions

In conclusion, 2SDT is a valid method for non-invasive characterization of LV wall motion in awake and anesthetized goats. In conjunction with conventional 2D, M-mode, and Doppler echocardiography, it may add to the understanding of LV mechanical function and may aid in the diagnosis of global and segmental LV systolic dysfunction in goats. However, effects of anesthesia and species-specific characteristics should be considered when goats are used as animal models for human disease. Furthermore, future studies are required to assess the reliability of 2DST measurements when used repeatedly in individual animals over time.

## Additional file

Additional file 1: Figure S5. Two-dimensional Speckle Tracking Analysis Tool. **Figure S6.** Two-dimensional Speckle Tracking Trace Screens. **Table S5.** Segmental 2DST analyses of left ventricular long-axis and short-axis recordings under awake and anesthetized conditions. **Table S6.** Segmental timing of peak strain in left ventricular long-axis and short-axis recordings under awake and anesthetized conditions.

## Abbreviations

2D: Two-dimensional
2DST: Two-dimensional speckle tracking
4CH: Four chamber view
Ant: Interventricular septal segment in short axis view
AntSept: Interventricular septal segment in short axis view
AVC_a_: Aortic valve closure (automatically determined)
AVC_m_: Aortic valve closure (manually determined)
D_L_: Longitudinal displacement
D_R_: Radial displacement
D_T_: Transverse displacement
ε: Strain
ε_C_: Circumferential strain
ε_C-sys_: Circumferential peak systolic strain
ε_sys_: Peak systolic strain
ECG: Electrocardiogram
EF: Ejection fraction
ε_L_: Longitudinal strain
ε_L-sys_: Longitudinal peak systolic strain
ε_R_: Radial strain
ε_R-sys_: Radial peak systolic strain
FS: Fractional shortening
HR_AVCa_: Instantaneous heart rate (for AVC_a_)
HR_AVCm_: Instantaneous heart rate (for AVC_m_)
Inf: Caudal LV free wall segment
Lat: Cranial LV free wall segment
LAX: Long axis
LV: Left ventricle or left ventricular
LVFW: Left ventricular free wall
LVOT: Left ventricular outflow tract
MAP: Mean arterial blood pressure
MV: Mitral valve
Post: Lateral LV free wall segment
PSM: Post-systolic motion
ROI: Region of interest
SAX: Short axis
SAX-AP: Short axis at apical level
SAX-CH: Short axis at chordal level
SAX-PM: Short axis at papillary muscle level
Sept: Interventricular septal segment in short axis view
SR: Strain rate
SR_C_: Circumferential strain rate
SR_C-A_: Circumferential peak late-diastolic strain rate
SR_C-E_: Circumferential peak early-diastolic strain rate
SR_C-sys_: Circumferential peak systolic strain rate
SR_L_: Longitudinal strain rate
SR_L-A_: Longitudinal peak late-diastolic strain rate
SR_L-E_: Longitudinal peak early-diastolic strain rate
SR_L-sys_: Longitudinal peak systolic strain rate
SR_R_: Radial strain rate
SR_R-A_: Radial peak late-diastolic strain rate
SR_R-E_: Radial peak early-diastolic strain rate
SR_R-sys_: Radial peak systolic strain rate
STIε: Synchrony time index
SV: Stroke volume
t_εC_: Time interval from the electrocardiographic R wave to circumferential peak strain
t_εL_: Time interval from the electrocardiographic R wave to longitudinal peak strain
t_εR_: Time interval from the electrocardiographic R wave to radial peak strain
t_AVCm_: Time of aortic valve closure (manually determined)
t_AVCa_: Time of aortic valve closure (automatically determined)
TDI: Tissue Doppler imaging

## References

[1] Boon JA (2010). Evaluation of size, function, and hemodynamics. Veterinary Echocardiography. 2nd edition.

[2] Otto CM (2004). Left and right ventricular systolic function. Textbook of Clinical Echocardiography.

[3] Teske AJ, Boeck BWD, Melman PG, Sieswerda GT, Doevendans PA, Cramer MJ (2007). Echocardiographic quantification of myocardial function using tissue deformation imaging, a guide to image acquisition and analysis using tissue Doppler and speckle tracking. Cardiovasc Ultrasound.

[4] Dandel M, Hetzer R (2009). Echocardiographic strain and strain rate imaging—clinical applications. Int J Cardiol.

[5] Edvardsen T, Gerber BL, Garot J, Bluemke DA, Lima JAC, Smiseth OA (2002). Quantitative assessment of intrinsic regional myocardial deformation by Doppler strain rate echocardiography in humans: validation against three-dimensional tagged magnetic resonance imaging. Circulation.

[6] Sutherland GR, Salvo GD, Claus P, D’hooge J, Bijnens B (2004). Strain and strain rate imaging: a new clinical approach to quantifying regional myocardial function. J Am Soc Echocardiogr.

[7] Marwick TH (2006). Measurement of strain and strain rate by echocardiography: ready for prime time?. J Am Coll Cardiol.

[8] Strain rate imaging: Cardiac deformation imaging by ultrasound/echocardiography–Tissue Doppler and speckle tracking. [http://folk.ntnu.no/stoylen/strainrate/] Last accessed: March 2015.

[9] Abraham TP, Dimaano VL, Liang H-Y (2007). Role of tissue Doppler and strain echocardiography in current clinical practice. Circulation.

[10] Lindqvist P, Borgström E, Gustafsson U, Mörner S, Henein MY (2009). Asynchronous normal regional left ventricular function assessed by speckle tracking echocardiography Appearances can be deceptive. Int J Cardiol.

[11] Cho GY, Marwick TH, Kim HS, Kim MK, Kyung-Soon H, Oh DJ (2009). Global 2-dimensional strain as a new prognosticator in patients with heart failure. J Am Coll Cardiol.

[12] Chetboul V, Serres F, Gouni V, Tissier R, Pouchelon JL (2007). Radial strain and strain rate by two-dimensional speckle tracking echocardiography and the tissue velocity based technique in the dog. J Vet Cardiol.

[13] Schwarzwald CC, Schober KE, Berli AS, Bonagura JD (2009). Left ventricular radial and circumferential wall motion analysis in horses using strain, strain rate, and displacement by 2D speckle tracking. J Vet Intern Med.

[14] Schefer KD, Bitschnau C, Weishaupt MA, Schwarzwald CC (2010). Quantitative analysis of stress echocardiograms in healthy horses with 2-dimensional (2D) echocardiography, anatomical M-mode, tissue Doppler imaging, and 2D speckle tracking. J Vet Intern Med.

[15] Decloedt A, Verheyen T, Sys S, De Clercq D, Van Loon G (2013). Two-dimensional speckle tracking for quantification of left ventricular circumferential and radial wall motion in horses. Equine Vet J.

[16] Decloedt A, Verheyen T, Sys S, De Clercq D, Van Loon G (2011). Quantification of left ventricular longitudinal strain, strain rate, velocity, and displacement in healthy horses by 2-dimensional speckle tracking. J Vet Intern Med.

[17] Smith MC, Sherman DM (2009). Cardiovascular System. Goat Medicine.

[18] Greiser M, Neuberger HR, Harks E, El-Armouche A, Boknik P, De Haan S (2009). Distinct contractile and molecular differences between two goat models of atrial dysfunction: AV block-induced atrial dilatation and atrial fibrillation. J Mol Cell Cardiol.

[19] Remes J, Van Brakel TJ, Bolotin G, Garber C, De Jong MM, Van Der Veen FH (2008). Persistent atrial fibrillation in a goat model of chronic left atrial overload. J Thorac Cardiovasc Surg.

[20] Van Brakel TJ, Hermans JJR, Accord RE, Schotten U, Smits JFM, Allessie MA (2009). Effects of intrapericardial sotalol and flecainide on transmural atrial electrophysiology and atrial fibrillation. J Cardiovasc Electrophysiol.

[21] Hallowell GD, Potter TJ, Bowen IM (2012). Reliability of quantitative echocardiography in adult sheep and goats. BMC Vet Res.

[22] Steininger K, Berli AS, Jud R, Schwarzwald CC (2011). Echocardiography in Saanen-goats: normal findings, reference intervals in awake goats, and the effect of general anesthesia. Schweiz Arch Tierheilkd.

[23] Aase SA, Støylen A, Ingul CB, Frigstad S, Torp H (2006). Automatic timing of aortic valve closure in apical tissue Doppler images. Ultrasound Med Biol.

[24] Voigt JU, Lindenmeier G, Exner B, Regenfus M, Werner D, Reulbach U (2003). Incidence and characteristics of segmental postsystolic longitudinal shortening in normal, acutely ischemic, and scarred myocardium. J Am Soc Echocardiogr.

[25] Opie LH (2004). Heart Physiology: From Cell to Circulation.

[26] Schwarzwald CC, Bonagura JD, Muir WW (2009). The cardiovascular system. Equine anesthesia-Monitoring and Emergency Therapy.

[27] Delgado V, Ypenburg C, Van Bommel RJ, Tops LF, Mollema SA, Marsan NA (2008). Assessment of left ventricular dyssynchrony by speckle tracking strain imaging comparison between longitudinal, circumferential, and radial strain in cardiac resynchronization therapy. J Am Coll Cardiol.

[28] Sengupta PP, Krishnamoorthy VK, Korinek J, Narula J, Vannan MA, Lester SJ (2007). Left ventricular form and function revisited: applied translational science to cardiovascular ultrasound imaging. J Am Soc Echocardiogr.

[29] Sengupta PP, Korinek J, Belohlavek M, Narula J, Vannan MA, Jahangir A (2006). Left ventricular structure and function: basic science in cardiac imaging. J Am Coll Cardiol.

[30] Saito K, Okura H, Watanabe N, Hayashida A, Obase K, Imai K (2009). Comprehensive evaluation of left ventricular strain using speckle tracking echocardiography in normal adults: comparison of three-dimensional and two-dimensional approaches. J Am Soc Echocardiogr.

[31] Sengupta PP, Khandheria BK, Korinek J, Wang J, Jahangir A, Seward JB (2006). Apex-to-Base dispersion in regional timing of left ventricular shortening and lengthening. J Am Coll Cardiol.

[32] Hamlin RL, Smith CR (1965). Categorization of common domestic mammals based upon their ventricular activation process. Ann N Y Acad Sci.

[33] Gorcsan J, Abraham T, Agler DA, Bax JJ, Derumeaux G, Grimm RA (2008). Echocardiography for cardiac resynchronization therapy: recommendations for performance and reporting–a report from the American Society of Echocardiography Dyssynchrony Writing Group endorsed by the Heart Rhythm Society. J Am Soc Echocardiogr.

[34] Voigt JU, Exner B, Schmiedehausen K, Huchzermeyer C, Reulbach U, Nixdorff U (2003). Strain-rate imaging during dobutamine stress echocardiography provides objective evidence of inducible ischemia. Circulation.

