# Supplementary material for: The use of strain, strain rate, and displacement by 2D speckle tracking for assessment of systolic left ventricular function in goats: applicability and influence of general anesthesia
Source: Cardiovasc Ultrasound. 2015 Mar 17;13:11. doi: 10.1186/s12947-015-0005-8 (PMC4373510; doi:10.1186/s12947-015-0005-8)
Supplement: Additional file 1: Figure S5. — Two-dimensional Speckle Tracking Analysis Tool. Figure S6. Two-dimensional Speckle Tracking Trace Screens. Table S5. Segmental 2DST analyses of left ventricular long-axis and short-axis recordings under awake and anesthetized conditions. Table S6. Segmental timing of peak strain in left ventricular long-axis and short-axis recordings under awake and anesthetized conditions. [file 12947_2015_5_MOESM1_ESM.pdf]

Additional file 1

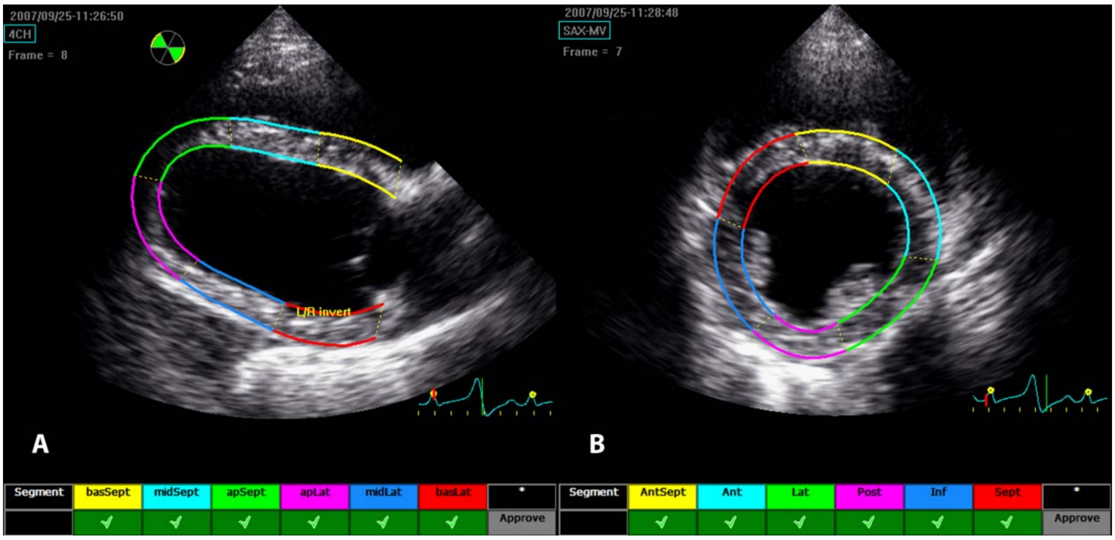

Figure S5 – Two-dimensional Speckle Tracking Analysis Tool

Two-dimensional speckle tracking analysis tool. The 2DST software automatically divides the region of interest into 6 segments. The quality of tracking is automatically verified by the software and segments with appropriate tracking are marked with a green ‘‘V’’ at the bottom of the screen.

A: Right-parasternal long-axis view of the left ventricle. The interventricular septum and the left ventricular free wall are divided into basal, mid-wall and apical segments.

B: Right-parasternal short-axis view at the level of the chordae tendineae. Note that the labels assigned to the short-axis segments are based on the human echocardiographic nomenclature and do not directly correspond to the anatomy of the goat heart. “Sept”, ‘‘AntSept’’ and ‘‘Ant’’ generally depict the interventricular septum, whereas ‘‘Lat’’, ‘‘Post’’ and ‘‘Inf’’ depict the cranial, lateral, and caudal LV free wall.

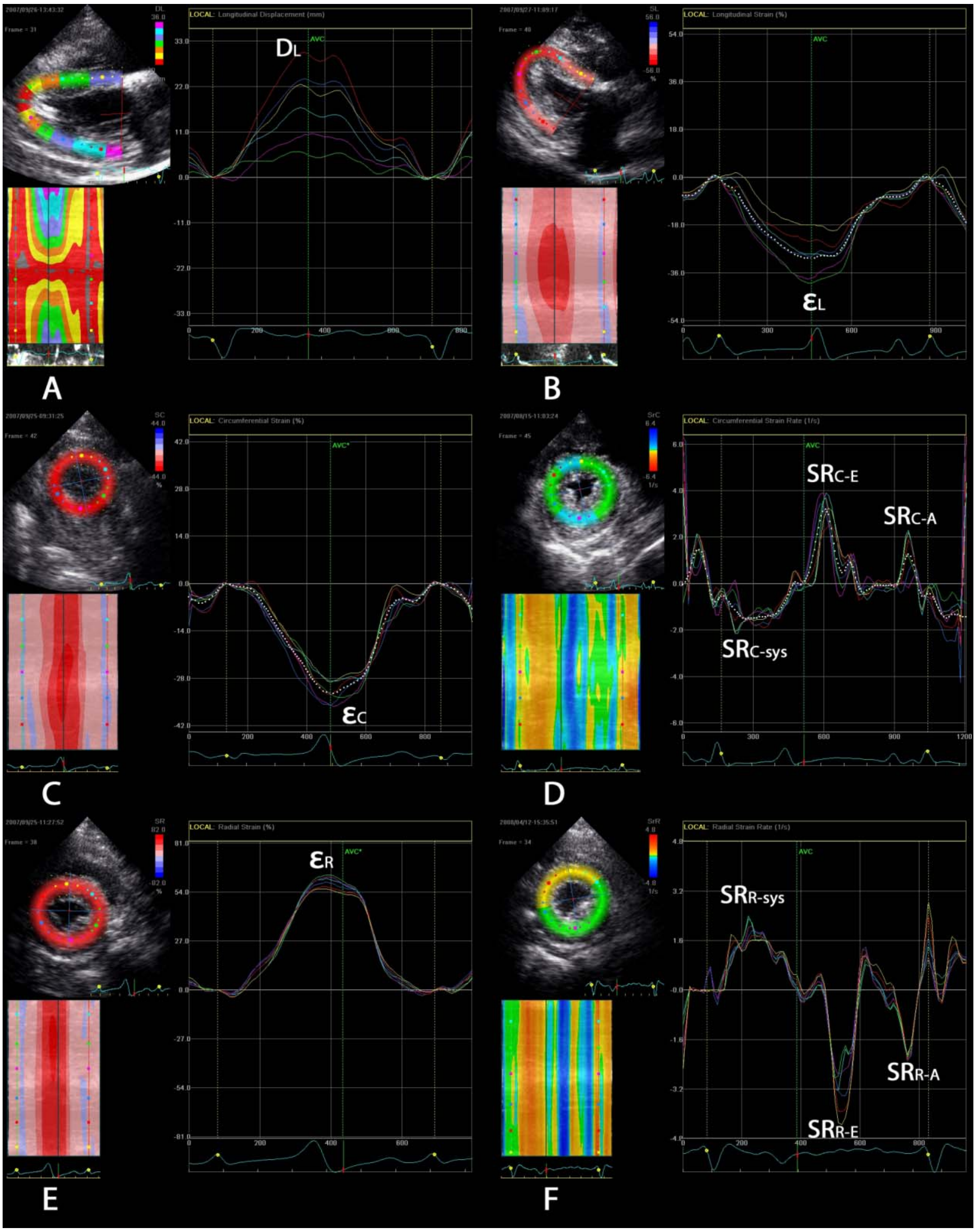

Figure S6 – Two-dimensional Speckle Tracking Trace Screens

Examples of trace screens of the 2DST analysis tool displaying the following information: Top left: 2D image with the segmented ROI and parametric color coding at the time of aortic valve closure. The center and the endocardial and epicardial borders of the different myocardial segments are marked by colored dots. Bottom left: M-mode with parametric color coding, showing the spatial (vertical axis) and temporal (horizontal axis) distribution of the respective measurements. The different

myocardial segments are marked by colored dots. An ECG is plotted for timing. Right: Trace display for the selected variable. The colors of the traces correspond to the colors of the segmented ROI. The dotted line (where shown) indicates the instantaneous average of all segments at the respective time of the cardiac cycle. An ECG is plotted for timing. The start and the end of the cycle are marked on the ECG with yellow dots. The automatically calculated time of aortic valve closure (AVC) is indicated by a green vertical line, dividing the cycle in its systolic and diastolic component.

A: Longitudinal displacement. D_L, longitudinal peak displacement. **B:** Longitudinal strain. ε_L, longitudinal peak strain. **C:** Circumferential strain. ε_c, circumferential peak strain. **D:** Circumferential strain rate. $SR_{C\text{-}sys}$, circumferential peak systolic strain rate; $SR_{C\text{-}E}$, circumferential peak early-diastolic strain rate; $SR_{C\text{-}A}$, circumferential peak late-diastolic strain rate. **E:** Radial strain. ε_R, radial peak strain. **F:** Radial strain rate. $SR_{R\text{-}sys}$, radial peak systolic strain rate; $SR_{R\text{-}E}$, radial peak early-diastolic strain rate; $SR_{R\text{-}A}$, radial peak late-diastolic strain rate.

Table S5 – Segmental 2DST analyses of left ventricular long-axis and short-axis recordings under awake and anesthetized conditions

			Segments						F-Test		
**Variable**	**Treatment**	**Units**	**basSept [mean ± SEM]**	**midSept [mean ± SEM]**	**apSept [mean ± SEM]**	**apLat [mean ± SEM]**	**midLat [mean ± SEM]**	**basLat [mean ± SEM]**	**p value Treatment**	**p value Segments**	**p value Treatment × Segments**
**LAX**											
$\varepsilon_{L\text{-sys}}$	Awake	%	-17.12 ± 0.64	-26.06 ± 0.64	-35.91 ± 0.64	-34.62 ± 0.64	-23.38 ± 0.64	-18.04 ± 0.68			
	Anesthetized		-17.22 ± 0.68	-26.48 ± 0.64	-36.99 ± 0.64	-35.04 ± 0.64	-24.68 ± 0.64	-20.43 ± 0.64			
									0.121	*<0.001*	0.548
ε_{L}	Awake	%	-18.78 ± 0.56	-26.49 ± 0.56	-36.02 ± 0.56	-34.84 ± 0.56	-25.11 ± 0.56	-22.31 ± 0.59			
	Anesthetized		-19.60 ± 0.59	-27.56 ± 0.56	-37.07 ± 0.56	-35.10 ± 0.56	-26.66 ± 0.56	-24.57 ± 0.56			
									0.022	*<0.001*	0.603
$SR_{L\text{-sys}}$	Awake	1/s	-1.64 ± 0.09	-2.09 ± 0.09	-2.85 ± 0.09	-2.81 ± 0.09	-2.03 ± 0.09	-2.06 ± 0.09			
	Anesthetized		-1.37 ± 0.09	-1.83 ± 0.09	-2.48 ± 0.09	-2.33 ± 0.09	-1.78 ± 0.09	-1.89 ± 0.09			
									<0.001	*<0.001*	0.451
$SR_{L\text{-E}}$	Awake	1/s	1.74 ± 0.12	2.24 ± 0.12	4.10 ± 0.12	4.12 ± 0.12	2.48 ± 0.12	2.77 ± 0.13			
	Anesthetized		1.51 ± 0.13	2.47 ± 0.12	4.42 ± 0.12	4.26 ± 0.12	2.75 ± 0.12	2.99 ± 0.12			
									0.323	*<0.001*	0.250
$SR_{L\text{-A}}$	Awake	1/s	1.53 ± 0.13	1.55 ± 0.13	2.08 ± 0.13	2.38 ± 0.13	1.76 ± 0.13	2.22 ± 0.13			
	Anesthetized		1.52 ± 0.13	1.24 ± 0.13	1.65 ± 0.13	1.72 ± 0.13	1.35 ± 0.13	1.58 ± 0.13			
									0.040	*<0.001*	0.135
DL	Awake	mm	20.90 ± 0.37	15.68 ± 0.37	6.65 ± 0.37	7.57 ± 0.37	18.55 ± 0.37	24.41 ± 0.37			
	Anesthetized		19.73 ± 0.37	14.55 ± 0.37	5.65 ± 0.42	8.91 ± 0.37	20.47 ± 0.37	27.15 ± 0.37			
									0.315	<0.001	*<0.001*
DT	Awake	mm	3.07 ± 0.26	4.54 ± 0.26	4.27 ± 0.26	3.97 ± 0.26	5.43 ± 0.26	8.76 ± 0.26			
	Anesthetized		3.44 ± 0.27	4.90 ± 0.26	4.85 ± 0.26	5.10 ± 0.26	7.03 ± 0.26	9.65 ± 0.26			
									0.011	*<0.001*	0.123

Variable	Treatment	Units	Segments						F-Test		
			**AntSept [mean ± SEM]**	**Ant [mean ± SEM]**	**Lat [mean ± SEM]**	**Post [mean ± SEM]**	**Inf [mean ± SEM]**	**Sept [mean ± SEM]**	**p value Treatment**	**p value Segments**	**p value Treatment × Segments**
**SAX-AP**											
$\varepsilon_{C\text{-}sys}$	Awake	%	-19.47 ± 0.90	-18.61 ± 0.90	-20.04 ± 0.90	-27.19 ± 0.90	-27.38 ± 0.90	-22.64 ± 0.90			
	Anesthetized		-24.82 ± 0.90	-20.12 ± 0.95	-19.94 ± 0.95	-26.34 ± 0.95	-28.97 ± 0.90	-28.34 ± 0.90			
									0.011	<0.001	*<0.001*
ε_{C}	Awake	%	-20.44 ± 0.88	-19.54 ± 0.88	-19.677 ± 0.88	-27.53 ± 0.88	-27.46 ± 0.88	-22.67 ± 0.88			
	Anesthetized		-25.15 ± 0.88	-21.33 ± 0.93	-20.78 ± 0.93	-26.87 ± 0.93	-29.05 ± 0.88	-28.48 ± 0.88			
									0.005	<0.001	*0.005*
$\varepsilon_{R\text{-}sys}$	Awake	%	52.02 ± 1.43	53.31 ± 1.43	55.77 ± 1.43	56.51 ± 1.43	56.07 ± 1.43	54.54 ± 1.43			
	Anesthetized		55.48 ± 1.43	55.28 ± 1.43	55.92 ± 1.43	55.24 ± 1.43	55.36 ± 1.43	55.56 ± 1.43			
									0.828	0.521	0.581
ε_{R}	Awake	%	56.54 ± 1.50	56.29 ± 1.50	58.03 ± 1.50	59.28 ± 1.50	59.65 ± 1.50	58.99 ± 1.50			
	Anesthetized		58.59 ± 1.50	57.51 ± 1.50	58.02 ± 1.50	58.69 ± 1.50	58.65 ± 1.50	58.97 ± 1.50			
									0.932	0.493	0.915
$SR_{C\text{-}sys}$	Awake	1/s	-1.75 ± 0.08	-1.89 ± 0.08	-1.94 ± 0.08	-2.51 ± 0.08	-2.53 ± 0.08	-2.20 ± 0.08			
	Anesthetized		-1.69 ± 0.08	-1.58 ± 0.08	-1.67 ± 0.08	-2.13 ± 0.08	-2.09 ± 0.08	-1.98 ± 0.08			
									0.013	*<0.001*	0.173
$SR_{C\text{-}E}$	Awake	1/s	2.90 ± 0.15	3.06 ± 0.15	2.88 ± 0.15	3.11 ± 0.15	2.61 ± 0.15	2.98 ± 0.15			
	Anesthetized		3.31 ± 0.15	3.10 ± 0.15	2.66 ± 0.16	3.59 ± 0.16	3.14 ± 0.15	3.45 ± 0.15			
									0.112	*0.010*	0.074
$SR_{C\text{-}A}$	Awake	1/s	1.41 ± 0.12	1.18 ± 0.12	1.07 ± 0.12	1.59 ± 0.12	1.81 ± 0.12	1.83 ± 0.12			
	Anesthetized		1.26 ± 0.12	0.80 ± 0.12	0.92 ± 0.12	0.96 ± 0.12	1.05 ± 0.12	1.50 ± 0.12			
									0.052	<0.001	*0.045*
$SR_{R\text{-}sys}$	Awake	1/s	2.21 ± 0.05	2.34 ± 0.05	2.39 ± 0.05	2.43 ± 0.05	2.40 ± 0.05	2.28 ± 0.05			
	Anesthetized		2.04 ± 0.05	2.11 ± 0.05	2.14 ± 0.05	2.19 ± 0.05	2.14 ± 0.05	2.12 ± 0.05			
									0.088	*0.009*	0.865
$SR_{R\text{-}E}$	Awake	1/s	-2.90 ± 0.11	-2.90 ± 0.11	-2.81 ± 0.11	-2.72 ± 0.11	-2.73 ± 0.11	-2.80 ± 0.11			
	Anesthetized		-2.91 ± 0.11	-2.71 ± 0.11	-2.82 ± 0.11	-2.90 ± 0.11	-2.82 ± 0.11	-2.88 ± 0.11			
									0.888	0.869	0.676
$SR_{R\text{-}A}$	Awake	1/s	-1.87 ± 0.06	-1.99 ± 0.06	-2.08 ± 0.06	-2.19 ± 0.06	-2.26 ± 0.06	-2.19 ± 0.06			
	Anesthetized		-1.34 ± 0.06	-1.32 ± 0.06	-1.35 ± 0.06	-1.32 ± 0.06	-1.40 ± 0.06	-1.41 ± 0.06			
									0.031	0.056	0.087
DR	Awake	mm	6.47 ± 0.26	7.73 ± 0.26	8.08 ± 0.26	7.56 ± 0.26	7.26 ± 0.26	6.71 ± 0.26			
	Anesthetized		6.98 ± 0.26	8.56 ± 0.26	10.41 ± 0.26	10.98 ± 0.26	9.06 ± 0.26	7.25 ± 0.26			
									<0.001	<0.001	*<0.001*

Variable	Treatment	Units	Segments						F-Test		
			**AntSept [mean ± SEM]**	**Ant [mean ± SEM]**	**Lat [mean ± SEM]**	**Post [mean ± SEM]**	**Inf [mean ± SEM]**	**Sept [mean ± SEM]**	**p value Treatment**	**p value Segments**	**p value Treatment × Segments**
**SAX-PM**											
$\varepsilon_{C\text{-}sys}$	Awake	%	-20.13 ± 0.99	-18.69 ± 1.17	-19.032 ± 1.11	-24.66 ± 0.99	-25.73 ± 0.99	-22.67 ± 0.99			
	Anesthetized		-28.59 ± 0.99	-21.46 ± 0.99	-16.60 ± 0.99	-19.24 ± 0.99	-25.60 ± 0.99	-28.94 ± 0.99	0.059	<0.001	*<0.001*
ε_{C}	Awake	%	-20.96 ± 0.93	-20.85 ± 1.10	-19.90 ± 1.04	-24.63 ± 0.93	-25.76 ± 0.93	-22.99 ± 0.93			
	Anesthetized		-28.64 ± 0.93	-22.37 ± 0.93	-17.85 ± 0.93	-20.24 ± 0.93	-25.82 ± 0.93	-29.77 ± 0.93	0.041	<0.001	*<0.001*
$\varepsilon_{R\text{-}sys}$	Awake	%	47.12 ± 1.53	52.67 ± 1.53	59.43 ± 1.53	61.11 ± 1.53	58.25 ± 1.53	52.72 ± 1.53			
	Anesthetized		52.81 ± 1.53	56.43 ± 1.53	63.92 ± 1.53	67.27 ± 1.53	67.67 ± 1.53	60.80 ± 1.53	0.122	*<0.001*	0.432
ε_{R}	Awake	%	50.59 ± 1.51	55.99 ± 1.51	62.08 ± 1.51	63.39 ± 1.51	60.30 ± 1.51	56.31 ± 1.51			
	Anesthetized		55.45 ± 1.51	59.10 ± 1.51	65.05 ± 1.51	69.28 ± 1.51	69.84 ± 1.51	64.55 ± 1.51	0.155	*<0.001*	0.169
$SR_{C\text{-}sys}$	Awake	1/s	-1.60 ± 0.08	-1.90 ± 0.10	-1.87 ± 0.09	-2.37 ± 0.08	-2.48 ± 0.08	-2.08 ± 0.08			
	Anesthetized		-1.89 ± 0.08	-1.64 ± 0.08	-1.40 ± 0.08	-1.79 ± 0.08	-2.18 ± 0.08	-2.03 ± 0.08	0.004	<0.001	*<0.001*
$SR_{C\text{-}E}$	Awake	1/s	2.08 ± 0.12	3.33 ± 0.14	3.08 ± 0.14	3.74 ± 0.12	2.89 ± 0.12	2.95 ± 0.12			
	Anesthetized		3.46 ± 0.12	2.95 ± 0.12	2.46 ± 0.12	3.53 ± 0.12	3.38 ± 0.12	3.33 ± 0.12	0.713	<0.001	*<0.001*
$SR_{C\text{-}A}$	Awake	1/s	1.22 ± 0.09	1.34 ± 0.10	1.12 ± 0.10	1.29 ± 0.09	1.26 ± 0.09	1.50 ± 0.09			
	Anesthetized		1.33 ± 0.09	1.07 ± 0.09	0.92 ± 0.09	0.89 ± 0.09	0.98 ± 0.09	1.24 ± 0.09	0.137	*0.034*	0.089
$SR_{R\text{-}sys}$	Awake	1/s	2.19 ± 0.07	2.57 ± 0.07	2.71 ± 0.07	2.63 ± 0.07	2.56 ± 0.07	2.30 ± 0.07			
	Anesthetized		2.08 ± 0.07	2.17 ± 0.07	2.16 ± 0.07	2.27 ± 0.07	2.34 ± 0.07	2.26 ± 0.07	0.016	<0.001	*0.002*
$SR_{R\text{-}E}$	Awake	1/s	-2.66 ± 0.07	-2.71 ± 0.07	-2.81 ± 0.07	-2.86 ± 0.07	-2.82 ± 0.07	-2.72 ± 0.07			
	Anesthetized		-2.64 ± 0.07	-2.68 ± 0.07	-2.89 ± 0.07	-3.02 ± 0.07	-2.90 ± 0.07	-2.71 ± 0.07	0.789	*0.011*	0.743
$SR_{R\text{-}A}$	Awake	1/s	-1.53 ± 0.13	-1.64 ± 0.13	-1.71 ± 0.13	-1.63 ± 0.13	-2.07 ± 0.13	-2.00 ± 0.13			
	Anesthetized		-1.23 ± 0.13	-1.28 ± 0.13	-1.35 ± 0.13	-1.40 ± 0.14	-1.55 ± 0.14	-1.58 ± 0.48	*0.045*	*0.006*	0.927
DR	Awake	mm	5.29 ± 0.29	6.04 ± 0.29	7.63 ± 0.29	8.70 ± 0.29	8.93 ± 0.29	7.41 ± 0.29			
	Anesthetized		5.64 ± 0.29	7.00 ± 0.29	10.66 ± 0.29	12.90 ± 0.29	11.72 ± 0.29	8.21 ± 0.29	<0.001	<0.001	*<0.001*

Variable	Treatment	Units	Segments						F-Test		
			**AntSept [mean ± SEM]**	**Ant [mean ± SEM]**	**Lat [mean ± SEM]**	**Post [mean ± SEM]**	**Inf [mean ± SEM]**	**Sept [mean ± SEM]**	**p value Treatment**	**p value Segments**	**p value Treatment × Segments**
**SAX-CH**											
$\varepsilon_{C\text{-}sys}$	Awake	%	-22.00 ± 1.01	-18.26 ± 1.07	-18.33 ± 1.22	-19.57 ± 1.07	-23.72 ± 1.01	-23.74 ± 1.01			
	Anesthetized		-29.82 ± 1.01	-22.47 ± 1.01	-16.77 ± 1.07	-19.10 ± 1.30	-25.03 ± 1.01	-30.46 ± 1.01	0.001	<0.001	*<0.001*
ε_{C}	Awake	%	-22.50 ± 1.04	-19.75 ±1.11	-19.31 ± 1.26	-19.69 ± 1.11	-23.47 ± 1.04	-29.94 ± 1.04			
	Anesthetized		-29.94 ± 1.04	-23.59 ± 1.04	-18.14 ± 1.11	-19.61 ± 1.34	-25.34 ± 1.04	-30.53 ± 1.04	0.001	<0.001	*<0.001*
$\varepsilon_{R\text{-}sys}$	Awake	%	40.07 ± 1.76	46.01 ± 1.76	58.08 ± 1.76	62.07 ± 1.76	60.27 ± 1.76	60.27 ± 1.76			
	Anesthetized		51.94 ± 1.76	53.09 ± 1.76	60.04 ± 1.76	65.99 ± 1.76	66.30 ± 1.76	61.42 ± 1.76	0.189	<0.001	*0.036*
ε_{R}	Awake	%	43.94 ± 1.66	50.07 ± 1.66	62.05 ± 1.66	66.20 ± 1.66	64.15 ± 1.66	56.56 ± 1.66			
	Anesthetized		55.01± 1.66	56.12 ± 1.66	63.97 ± 1.66	69.73 ± 1.66	70.08 ± 1.66	65.23 ± 1.66	0.17	*<0.001*	0.083
$SR_{C\text{-}sys}$	Awake	1/s	-1.81 ± 0.07	-1.85 ± 0.08	-1.97 ± 0.09	-2.33 ± 0.08	-2.62 ± 0.07	-2.08 ± 0.07			
	Anesthetized		-1.96 ± 0.07	-1.73 ± 0.07	-1.45 ± 0.08	-1.69 ± 0.09	-1.92 ± 0.07	-2.00 ± 0.07	0.005	<0.001	*<0.001*
$SR_{C\text{-}E}$	Awake	1/s	2.90 ± 0.14	2.88 ± 0.15	3.04 ± 0.17	3.60 ± 0.15	3.48 ± 0.14	2.72 ± 0.14			
	Anesthetized		3.61 ± 0.14	3.20 ± 0.14	2.42 ± 0.15	3.46 ± 0.18	3.76 ± 0.14	3.42 ± 0.14	0.259	<0.001	*<0.001*
$SR_{C\text{-}A}$	Awake	1/s	1.36 ± 0.09	1.33 ± 0.10	1.37 ± 0.11	1.23 ± 0.10	1.20 ± 0.09	1.46 ± 0.09			
	Anesthetized		1.30 ± 0.09	1.06 ± 0.09	0.90 ± 0.09	1.11 ± 0.11	1.11 ± 0.09	1.37 ± 0.09	0.214	0.147	0.296
$SR_{R\text{-}sys}$	Awake	1/s	2.25 ± 0.07	2.52 ± 0.07	2.71 ± 0.07	2.72 ± 0.07	2.76 ± 0.07	2.50 ± 0.07			
	Anesthetized		2.19 ± 0.07	2.14 ± 0.07	2.32 ± 0.07	2.39 ± 0.07	2.48 ± 0.07	2.43 ± 0.07	0.213	*<0.001*	0.05
$SR_{R\text{-}E}$	Awake	1/s	-2.75 ± 0.10	-2.68 ± 0.10	-2.97 ± 0.10	-3.27 ± 0.10	-3.34 ± 0.10	-3.10 ± 0.10			
	Anesthetized		-2.79 ± 0.10	-2.70 ± 0.10	-2.73 ± 0.10	-2.88 ± 0.10	-2.89 ± 0.10	-2.76 ± 0.10	0.318	*<0.001*	0.055
$SR_{R\text{-}A}$	Awake	1/s	-1.58 ± 0.09	-1.52 ± 0.09	-1.74 ± 0.09	-1.90 ± 0.09	-1.92 ± 0.09	-1.83 ± 0.09			
	Anesthetized		-1.35 ± 0.09	-1.48 ± 0.09	-1.66 ± 0.09	-1.68 ± 0.09	-1.76 ± 0.09	-1.66 ± 0.09	0.451	*<0.001*	0.894
DR	Awake	mm	4.08 ± 0.26	4.13 ± 0.26	7.12 ± 0.26	9.68 ± 0.26	9.95 ± 0.26	7.59 ± 0.27			
	Anesthetized		4.83 ± 0.26	6.03 ± 0.26	10.21 ± 0.26	13.49 ± 0.26	12.39 ± 0.26	8.55 ± 0.26	<0.001	<0.001	*<0.001*

$\varepsilon_{L\text{-}sys}$ longitudinal peak systolic strain; ε_L, longitudinal peak strain; $SR_{L\text{-}sys}$, longitudinal peak systolic strain rate; $SR_{L\text{-}E}$, longitudinal peak early-diastolic strain rate; $SR_{L\text{-}A}$, longitudinal peak late-diastolic strain rate; DL, longitudinal peak displacement; DT, transverse peak displacement; $\varepsilon_{C\text{-}sys}$, circumferential peak systolic strain; ε_C, circumferential peak strain; $\varepsilon_{R\text{-}sys}$, radial peak systolic strain; ε_R, radial peak strain; $SR_{C\text{-}sys}$, circumferential peak systolic strain rate; $SR_{C\text{-}E}$, circumferential peak early-diastolic strain rate; $SR_{C\text{-}A}$, circumferential peak late-diastolic strain rate; $SR_{R\text{-}sys}$, radial peak systolic strain rate; $SR_{R\text{-}E}$, radial peak early-diastolic strain rate; $SR_{R\text{-}A}$, radial peak late-diastolic strain rate; DR, radial peak displacement.

Table S6 – Segmental timing of peak strain in left ventricular long-axis and short-axis recordings under awake and anesthetized conditions

			Segments						F-Test		
**Variable**	**Treatment**	**Unit**	**basSept [mean ± SEM]**	**midSept [mean ± SEM]**	**apSept [mean ± SEM]**	**apLat [mean ± SEM]**	**midLat [mean ± SEM]**	**basLat [mean ± SEM]**	**p value Treatment**	**p value Segments**	**p value Treatment × Segments**
**LAX**											
$t_{\varepsilon L}$	Awake	ms	318 ± 5	297 ± 5	278 ± 5	286 ± 5	321 ± 5	338 ± 5			
	Anesthetized		373 ± 5	360 ± 5	326 ± 5	317 ± 5	372 ± 5	389 ± 5			
									<0.001	*<0.001*	0.052
			**Segments**								
			**AntSept [mean ± SEM]**	**Ant [mean ± SEM]**	**Lat [mean ± SEM]**	**Post [mean ± SEM]**	**Inf [mean ± SEM]**	**Sept [mean ± SEM]**			
**SAX-AP**											
$t_{\varepsilon C}$	Awake	ms	311 ± 6	314 ± 6	283 ± 6	281 ± 6	270 ± 6	293 ± 6			
	Anesthetized		337 ± 6	368 ± 7	355 ± 7	342 ± 7	322 ± 6	323 ± 6			
									<0.001	<0.001	*0.001*
$t_{\varepsilon R}$	Awake	ms	314 ± 5	296 ± 5	292 ± 5	301 ± 5	307 ± 5	313 ± 5			
	Anesthetized		352 ± 5	349 ± 5	347 ± 5	347 ± 5	354 ± 5	371 ± 5			
									<0.001	*0.001*	0.445
**SAX-PM**											
$t_{\varepsilon C}$	Awake	ms	303 ± 6	338 ± 7	298 ± 6	282 ± 6	277 ± 6	290 ± 6			
	Anesthetized		328 ± 6	365 ± 6	370 ± 6	338 ± 6	331 ± 6	323 ± 6			
									<0.001	<0.001	*<0.001*
$t_{\varepsilon R}$	Awake	ms	309 ± 4	302 ± 4	290 ± 4	289 ± 4	292 ± 4	304 ± 4			
	Anesthetized		339 ± 4	337± 4	344 ± 4	350 ± 4	355 ± 4	347 ± 4			
									<0.001	0.075	*<0.001*
**SAX-CH**											
$t_{\varepsilon C}$	Awake	ms	297 ± 6	339 ± 7	334 ± 8	294 ± 7	282 ± 6	294 ± 6			
	Anesthetized		326 ± 6	361 ± 6	366 ± 7	343 ± 8	341 ± 6	323 ± 6			
									<0.001	<0.001	*0.045*
$t_{\varepsilon R}$	Awake	ms	329 ± 5	320 ± 5	313 ± 5	315 ± 5	319 ± 5	330 ± 5			
	Anesthetized		355 ± 5	355 ± 5	361 ± 5	367 ± 5	366 ± 5	363 ± 5			
									0.078	0.484	0.112

LAX, long axis view; SAX-AP, short axis view at apical level; SAX-PM, short axis view at papillary muscle level; SAX-CH, short axis view at chordal level; $t_{\varepsilon L}$, Time to peak longitudinal strain; $t_{\varepsilon C}$, Time to peak circumferential strain; $t_{\varepsilon R}$, Time to peak radial strain.
